# Antithrombotic drugs for carotid artery dissection: Updated systematic review

**DOI:** 10.1177/23969873241292278

**Published:** 2024-10-26

**Authors:** Nikolaos S Avramiotis, Fabian Schaub, Sebastian Thilemann, Philippe Lyrer, Stefan T Engelter

**Affiliations:** 1 Department of Neurology and Stroke Center, University Hospital Basel, Basel, Switzerland; 2 Department of Clinical Research, University of Basel, Basel, Switzerland; 3 Department of Neurology, Inselspital, University Hospital and University of Bern, Bern, Switzerland; 4 Neurology and Neurorehabilitation, University Department of Geriatric Medicine FELIX PLATTER, University of Basel, Basel, Switzerland

**Keywords:** Dissection, carotid, antiplatelets, anticoagulants, death, disability, stroke

## Abstract

**Introduction:**

Extracranial internal carotid artery dissection (eICAD) is a leading cause of stroke in younger patients. In this Cochrane Review update we compared benefits and harms of eICAD-patients treated with either antiplatelets or anticoagulants.

**Patients and methods:**

Eligible studies were identified through Cochrane Stroke Group Trials Register, CENTRAL, MEDLINE, and EMBASE and personal search until December 2023. We included randomized-controlled trials (RCTs) and non-randomized studies comparing anticoagulants with antiplatelets in eICAD-patients. Co-primary outcomes were (i) death (all causes) and (ii) death or disability. Secondary outcomes were ischemic stroke, symptomatic intracranial hemorrhage, and major extracranial hemorrhage. Odds ratios (OR) with 95% CIs were calculated for (i) all studies and (ii) separately for RCTs and non-randomized studies.

**Results:**

We meta-analyzed a total of 42 studies (2624 patients) including 2 RCTs (213 patients) for the primary outcome of death and 31 studies (1953 patients) including 1 RCT (115 patients) for the primary outcome of death or disability. Antiplatelet-treated patients had higher odds for death (OR_all-studies_ 2.70, 95% CI 1.27–5.72; OR_RTCs_ 6.80, 95% CI 0.14–345; OR_non-randomized studies_ 2.60, 95% CI 1.20–5.60) and death or disability (OR_all-studies_ 2.1, 95% CI 1.58–2.66; OR_RTCs_ 2.2, 95% CI 0.29–16.05; OR_non-randomized studies_ 2.1, 95% CI 1.58–2.66) than anticoagulated patients. Antiplatelet-treated patients had also higher odds for ischemic stroke, though this reached statistical significance only in the subgroup of RCTs (OR_RTC_ 4.60, 95% CI 1.36–15.51). In turn, antiplatelet-treated patients had less symptomatic intracranial hemorrhage (OR_all-studies_ 0.25, 95% CI 0.07–0.86) and a tendency toward less major extracranial hemorrhage (OR_all-studies_ 0.17, 95% CI 0.03–1.03).

**Discussion and conclusion:**

The evidence considering antiplatelets as standard of care in eICAD is weak. Individualized treatment decisions balancing risks versus harms seem recommendable.

## Graphical abstract

**Graphical abstract:**
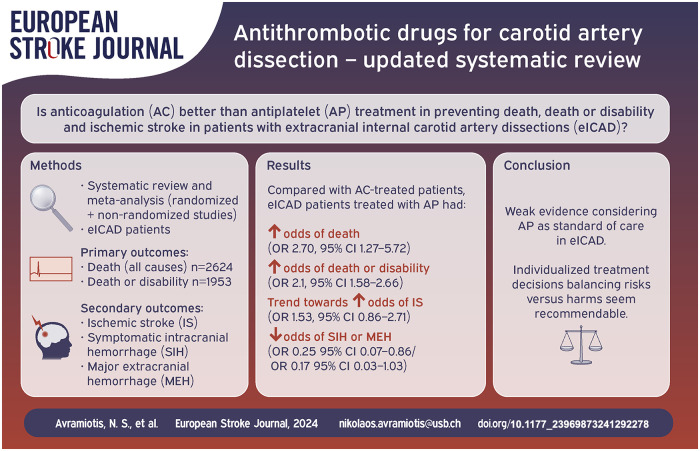

## Introduction

Extracranial (cervical) internal carotid artery dissection (eICAD) is a leading cause of stroke in young patients (up to 25% in patients <50 years)^1–3^ but also occurs in subjects aged 60 years and older.^4^

Antithrombotic medication for stroke prevention is the mainstay of management of patients with eICAD.^5^ However, it is still unclear whether to use antiplatelets (AP) or anticoagulation (AC), as superiority of either treatment modality is still to be proven. Two recently published randomized controlled clinical trials comparing AP to AC in cervical artery dissections (CADISS and TREAT-CAD),^6,7^ as well as meta-analyses on the study level^8^ and based on individual-patient data of both trials^9^ did not find superiority of either approach. However, both randomized trials and two recent meta-analyses^8,10^ did not stratify their findings in patients with ICAD versus those with VAD.^6–8,11^

The approach chosen for this research is different to that of the aforementioned meta-analyses as it focused on the antithrombotic treatment response solely for ICAD (rather than for cervical artery dissection – as the combination of VAD and ICAD). This approach takes into account that ICAD differs from VAD in several aspects. Compared to VAD patients, ICAD patients were predominantly male^12^ and older,^4,13^ had a recent infection more often,^14^ presented with stroke^13,15^ or with occlusive dissection^16^ less often, have more severe strokes,^13,15^ and a poor functional outcome.^17^ Furthermore, ICA originates from the neural crest, while VA originates from the mesoderm.^13^

In addition, since the last update, the use of both dual antiplatelets as well as direct oral anticoagulants has been employed^8,18,19^ in eICAD. The objective of this updated systematic review, using the Cochrane methodology, was to evaluate the current evidence about benefits and harms of anticoagulants (AC) versus antiplatelets (AP) in patients with eICAD.

## Methods

The protocol for this systematic review was first published in Cochrane Database of Systematic Reviews Issue 4, 1998; CD Number: CD000255.^20^ There have been no amendments to the protocol. This systematic review was performed according to the PRISMA (Preferred Reporting Items for Systematic Reviews and Meta-Analysis) guidelines.^21^ Data not published within the article are available from the corresponding author upon reasonable request.

### Eligibility criteria

All randomized controlled trials (RCTs), controlled clinical trials (CCTs), non-randomized studies including case series with at least four patients with symptomatic eICAD that allowed comparisons between antithrombotic treatments for eICAD were eligible. We included all studies that reported at least one primary outcome comparing patients treated with AC versus those treated with AP. In line with prior versions of this review,^20,22,23^ studies reporting on a single treatment modality, including three or fewer cases, consisting of reviews summarizing case reports, series with data repetition from former citations (we included only the most recent series) or studies where we could not make the distinction between dissections of internal carotid artery, common carotid artery, vertebral artery, or intracranial carotid artery dissection were excluded.

Patients’ symptoms included: stroke, transient ischemic attacks or pure local neurological deficits. Studies with patients whose diagnosis of eICAD was made by arterial angiography, duplex scanning, computer tomography, or magnetic resonance imaging demonstrating specific features of dissection were eligible. We accepted the following angiographic signs consistent with eICAD: mural hematoma, dissecting aneurysm, long tapering stenosis, intimal flap, double lumen, or occlusion more than 2 cm above the carotid bifurcation revealing a dissecting aneurysm or a long tapering stenosis after recanalization, applying widely accepted criteria.^13,24^

Antithrombotic treatment was defined as administration of any AP drug or combinations thereof (i.e. acetylsalicylic acid (ASA), ticlopidine, clopidogrel, sulfinpyrazone, dipyridamole, ticagrelor, prasugrel) or administration of full dose AC (such as intravenous or subcutaneous fractionated or unfractionated heparin and/or oral coumarin or DOACS).^25^

We only analyzed the initially used antithrombotic treatment and excluded the patients from the case series for whom surgical intervention or stenting was mentioned as a treatment modality.

In patients receiving thrombolysis and/or thrombectomy, the first antithrombotic agent used thereafter was deemed the “initially used antithrombotic treatment.” In cases of bleeding complications among such patients, we sought information on whether the bleed was associated with the thrombolytic/thrombectomy treatment (rather than with the antithrombotic treatment).

### Outcomes

Co-primary outcomes were^22^: “death from all causes” and “death or disability” (defined according to the modified Rankin Scale (mRS) as mRS ⩾ 3 at the end of the follow-up period).

In the studies where disability was not defined, we assessed the outcome based on the clinical information mentioned in the publications as done in prior versions of the review.^22^

Secondary outcomes were^22^: Ischemic stroke - according to the WHO definition^26^ including retinal infarctions - occurring under antithrombotic therapy.

Symptomatic intracranial hemorrhage according to the definition used in the individual study. If no definition was given, we considered any neurological worsening associated with intracranial blood visible on neuroimages as a symptomatic intracranial hemorrhage.^22^

Major extracranial hemorrhage as defined in the individual study. If no definition was given, we considered any clinically apparent extracranial bleeding resulting in a surgical or endoscopic intervention or a transfusion a major extracranial hemorrhage.^22^

### Search

Eligible studies were identified through Cochrane Stroke Group Trials Register, CENTRAL, MEDLINE, and EMBASE and personal search up to December 2023. The used search strategies can be found in the Supplemental Material (Tables S1–S3).

In addition, we screened reference lists of relevant recent review articles and primary studies found for additional eligible studies (including recent review papers^8,25^), contacted authors and experts in the field to identify further published or unpublished trials, contacted the main authors of studies if data reported in the original articles were incomplete (Email or personal communication) and searched ClinicalTrials.gov (http://clinicaltrials.gov/) for further trials.

### Data extraction

NSA, ST, and FS screened titles and abstracts from the list retrieved by the search process and selected the studies which met the eligibility criteria. NSA and ST individually extracted all outcome measures from the selected studies, independently from each other. In any case of missing information or uncertainties, study authors were contacted through email. Any disagreements or discrepancies were independently reviewed by a senior author (PL or STE) and were resolved by discussion within the author team.

### Risk of bias and certainty of evidence

The risk of bias was assessed by LP, NSA and STE for (i) randomized studies with the Risk of Bias tool of the Cochrane Handbook for Systematic Reviews of Interventions,^27^ which distinguished of low, high, or unclear risk for the following bias domains: selection, performance, detection, attrition, reporting and other sources of bias. (ii) For all non-randomized studies the risk of confounding, selection, information and reporting bias was evaluated. In addition, publication bias was assessed by visual inspection of funnel plots for each outcome.^28^ The same raters also used the GRADE approach to determine the certainty of evidence for each of the five outcomes using the five GRADE considerations (risk of bias, consistency of effect, imprecision, indirectness, and publication bias). In case of disagreements, consensus was reached by discussion. GRADE assessment (GRADEPro GDT) can be found in the Supplemental Material (Table S4).

### Data analysis and post-hoc sensitivity analysis

We performed the data analysis using RevMan Web. We calculated a weighted estimate of the odds for each outcome event across studies using the Peto odds ratio method. We calculated ORs with 95% CIs using the Peto fixed-effect method^29^ as done in the prior versions of this review.^20,22^ We assessed the heterogeneity between trial results using the I2 statistic.^30^ We report on (i) all studies but (ii) also distinguished randomized trials from non-randomized studies.

Post-hoc we performed the following sensitivity analyses regarding the outcomes of “death,” “death or disability,” and “ischemic stroke” (i) excluding all case series and observational studies without consecutive patients included, (ii) excluding smaller studies with (a) <50 patients or (b) <100 patients, (iii) excluding older studies (i.e. published before 2007), and (iv) excluding studies of patients with traumatic ICAD. Furthermore, post-hoc we also dichotomized the included studies based on length of follow-up duration that is, follow-up at 3 or 6 months, versus follow-up at 1 year or more (mean). Studies with information only concerning the initial hospitalization or with unknown follow-up timing were excluded.

## Results

**Figure 1.**
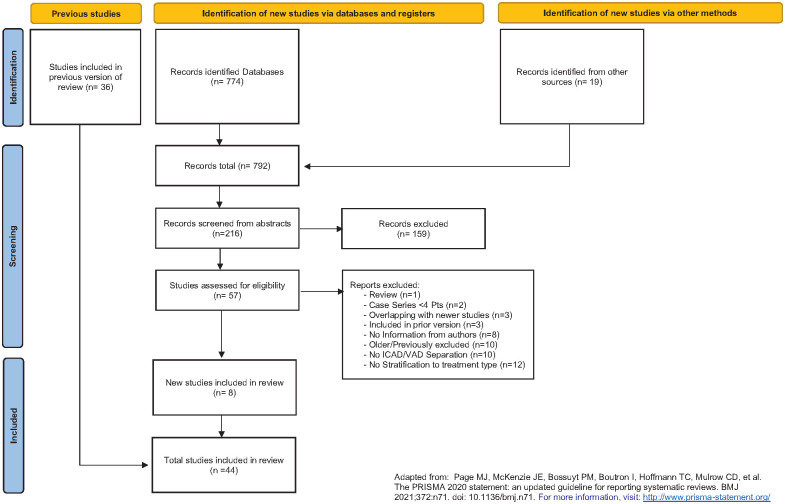
Flow chart for included studies and reasons for exclusion.

The new search yielded 2 completed randomized trials, CADISS and TREAT-CAD^6,7^ and 6 new non-randomized studies.^31–36^ Together with the previously included 36 studies this review is based on a total of 44 studies (Figure 1), of which 42 studies (death) and 31 studies (death or disability), respectively provided data on the co-primary outcomes while 2 studies^37,38^ provided data only on the secondary outcomes. Details about each individual included study, including duration of follow-up and sources of data can be found in the Supplemental Material (Tables S5 and S6). Forty-nine (49) studies were excluded, the reasons for each exclusion are shown in the review’s flow chart (Figure 1) and a complete comprehensive table of all excluded studies can be found in the Supplemental Material (Table S7).

### Risk of bias

**Figure 2.**
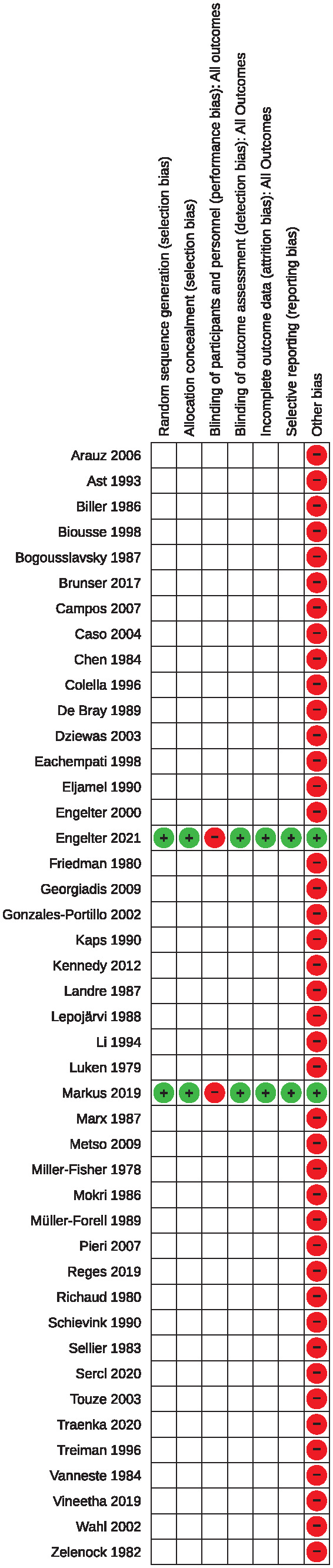
Risk of bias in the included studies.

The risk of bias of both RCTs was considered low in all categories with the exception of performance bias. Due to the absent blinding to the allocated treatment, the risk of performance bias was considered high (Figure 2).

For all non-randomized studies, the risk of bias was rated serious as all were observational, retrospective and none reported results from a pre-defined treatment protocol. Furthermore, the choice whether to use antiplatelet or anticoagulant treatment was decided by the treating physician and/or the patients, leading to a serious risk of allocation and selection bias. Even though an effort was done in most studies to minimize confounders, we assume that preference of patients (e.g. for AP rather than AC) and personal experience of physicians (e.g. bleed under AC in severely affected patients) as well as difference in cost (AC is usually more expensive than AP) may act as confounding factors. Furthermore, the length of follow up was heterogeneous among studies and varied within studies. Finally, due to absence of pre-defined and standardized procedures about screening, inclusion and treating patients and about the mode of outcome assessment, we assume that the risk of a reporting bias is substantial. As we do not consider the risk of bias to be on a critical level (all studies were published in peer-reviewed journals), all selected non-randomized studies were included in the analysis.

### Primary outcomes

#### Death from all causes

**Figure 3.**
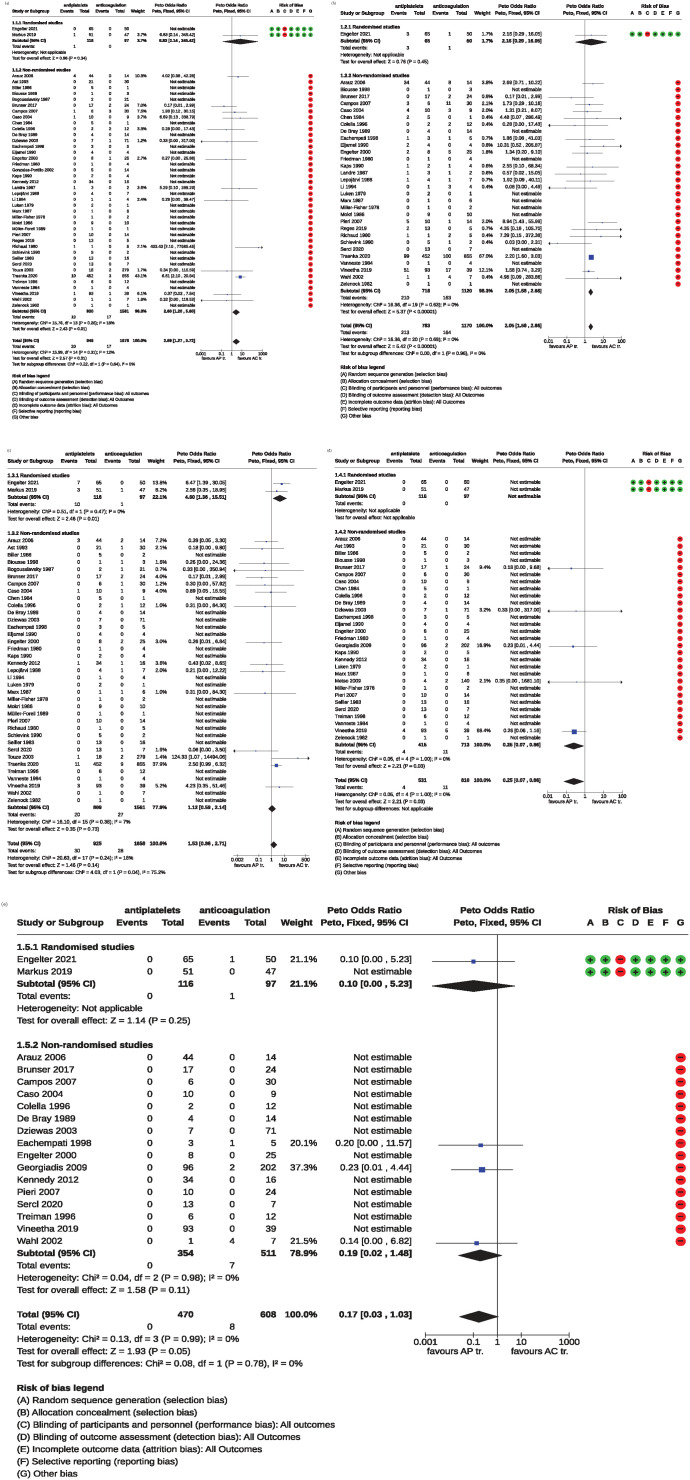
Forrest plots of the Meta-analyses comparing APs to ACs. Estimates to the left of the vertical line favor APs and those to the right favor ACs: (a) death from all causes, (b) death or disability, (c) ischemic stroke, (d) symptomatic intracranial hemorrhage, and (e) major extracranial hemorrhage.

For the outcome death from all causes, we analyzed data from 42 studies (2 randomized and 40 non-randomized) with 2624 patients. In total, 37 of 2624 (1.4%) patients were reported dead at the end of follow up, with 20/946 (2.1%) in the AP-group and 17/1678 (1%) in the AC-group. The Peto odds ratio of 2.7 with a 95% CI ranging from 1.27 to 5.72 (*p* = 0.01) showed an overall benefit of anticoagulation with respect to death during the follow-up period. There was no significant heterogeneity between the included series (*I*^2^ = 12%; Figure 3(a)).

For the subgroup of RCTs, data from 2 trials with 213 ICAD patients were analyzed with 1 of 213 (0.4%) patients reported dead at the end of follow up, 1/116 in the AP and 0/97 in the AC group, amounting to a Peto odds ratio of 6.8 with a 95% CI ranging from 0.14 to 345.42 (*p* = 0.34).

For the subgroup of non-randomized studies (*n* = 40, 2411 patients), in total, 36 of 2411 (1.49%) patients were reported dead at the end of follow up. The Peto odds ratio of 2.60 with a 95% CI ranging from 1.20 to 5.60 (*p* = 0.01) indicated a significant benefit of anticoagulation with respect to death during the follow-up period. There was a non-significant heterogeneity between the included studies (*I*^2^ = 18%; Figure 3(a)).

### Death or disability

For the outcome death or disability, based on 31 studies (1953 patients) a benefit of anticoagulants was obtained (OR 2.1, 95% CI 1.58–2.66; *p* < 0.00001). There was no significant heterogeneity between the included series (*I*^2^ = 0%) (Figure 3(b)).

For the subgroup of RCTs, the analysis was based on 1 trial (115 patients). There were 4 of 115 patients reported dead or disabled at the end of follow up, 3/65 (4.6%) in the AP and 1/50 (2%) in the AC-group, respectively, amounting to a Peto OR of 2.2 (95% CI 0.29–16.05; *p* = 0.45).

For the subgroup of non-randomized studies (30 studies; 1838 patients), the Peto OR of 2.1 (95% CI 1.58–2.66; *p* < 0.00001), showed a benefit of anticoagulants (Figure 3(b)).

### Secondary outcomes

#### Ischemic stroke

Thirty-nine studies with 2583 patients reported on ischemic strokes at the end of the follow-up period. In 58 patients (2.24%) ischemic strokes were recorded. The stroke rate in the anticoagulation group was 1.7% (28/1658) and 3.2% (30/925) in the AP group, amounting to a Peto odds ratio of 1.53 (95% CI 0.86–2.71, *p* = 0.14). There was a non-significant heterogeneity between all included studies (*I*^2^ = 18%; Figure 3(c)).

In the subgroup of RCTs (2 trials, 213 patients), in 11 patients (5.2%) ischemic strokes were recorded. The stroke rate in the anticoagulation group was 1.0% (1/97) and 8.6% (10/116) in the AP group, respectively, yielding to a Peto odds ratio of 4.60 (95% CI 1.36–15.51, *p* = 0.01) in favor of anticoagulants.

For the subgroup of non-randomized studies (37 studies, 2370 patients), in 47 patients (2.0%) ischemic strokes were recorded. The stroke rate in the anticoagulation group was 1.7% (27/1561) and 2.5% (20/809) in the AP group resulting in a Peto odds ratio of 1.12 (95% CI 0.59–2.14, *p* = 0.73; Figure 3(c)).

### Symptomatic intracranial hemorrhage

Thirty-one studies with 1341 patients provided data about symptomatic intracranial hemorrhage stratified on the type of antithrombotic treatment. Symptomatic intracranial hemorrhages were present in 15 of 1341 patients (1.1%), 4/531 (0.8%) in the AP and in 11/810 (1.4%) in the AC group. The Peto odds ratio of 0.25 with a 95% CI of 0.07–0.86 indicated a significant difference between the treatment options in favor of the AP group (*p* = 0.03). There was no significant heterogeneity between the included studies (*I*^2^ = 0%; Figure 3(d)).

In the subgroup of RCTs (2 trials, 213 patients), no symptomatic intracranial hemorrhages occurred.

For the subgroup of non-randomized studies (29 studies, 1128 patients), symptomatic intracranial hemorrhages were present in 15 of 1128 patients (1.3%). The Peto odds ratio of 0.25 with a 95% CI of 0.07–0.86 indicated a difference between the treatment options in favor of the AP group (*p* = 0.03; Figure 3(d)).

### Major extracranial hemorrhage

Analysis in respect of major extracranial hemorrhage was based on 18 studies with 1078 patients. Major extracranial hemorrhages occurred only in the AC group and were present in 8 of 608 patients (1.3%) The Peto odds ratio of 0.17 with a 95% CI of 0.03–1.03 indicated a just not statistically significant difference between the treatment options (*p* = 0.05) with a tendency toward a favorable effect for AP. There was no significant heterogeneity between the included series (*I*^2^ = 0%; Figure 3(e)).

In the subgroup of RCTs, major extracranial hemorrhages occurred only in the AC group and were present in 1 of 97 patients (1.0%). The Peto odds ratio of 0.10 with a 95% CI of 0.00–5.23 indicated no significant difference between the treatment options (*p* = 0.25).

For the subgroup of non-randomized studies (16 studies, 865 patients), major extracranial hemorrhages occurred only in the AC group and were present in 7 of 511 patients (1.4%). The Peto odds ratio of 0.19 with a 95% CI of 0.02–1.48 indicated no significant difference between the treatment options (*p* = 0.11; Figure 3(e)).

### Post-hoc sensitivity analyses

The post-hoc sensitivity analyses excluding (i) case series and observational without consecutive patients included (*n* = 11 studies), (ii) smaller studies with (a) less than 50 or (b) less than 100 patients (*n* = 33 and 37 studies, respectively), or (iii) older studies (*n* = 32 studies), or (iv) studies of patients with traumatic ICAD (*n* = 7 studies) did not result in any substantial differences to the results obtained in the primary analyses. For the outcome “ischemic stroke,” excluding smaller studies as well as older studies even showed a statistically significant difference in favor of AC compared to AP.

The post-hoc sensitivity analysis with a dichotomized length of follow-up duration (i.e. 3/6 months vs 1 year and longer) showed that across studies with a 3/6month follow-up there was a statistically significant difference in favor of the AC group for the outcomes “death,” “death or disability,” and “ischemic stroke,” which was not the case across studies with a follow-up duration of 1 year and more. All results are shown in detail in the Supplemental Material (Figures S1–S7).

## Discussion

This updated systematic review indicates a significant benefit of anticoagulants over antiplatelets for the co-primary outcomes “death from all causes” based on 2624 patients and “death or disability” based on 1953 patients, 213 and 115 of them derived from randomized trials respectively. The frequency of death was approximately double in AP-treated than among AC-treated patients albeit with a wide confidence interval indicating that the difference could be smaller or larger. Interestingly, even though the frequency in both groups was different – AP about 2% AC about 1% – it was low, and certainly lower than originally suggested.^39,40^ However, some patients with severe infarctions were excluded from several studies because they had received neither anticoagulants nor antiplatelet agents^38,41–44^ had malignant infarctions^38^ or were treated with stenting.^34^ In another included study patients with stroke at presentation were excluded.^36^ Therefore, the estimated death rate of this review reflects not that of eICAD patients in general but that of patients who are well enough to receive any kind of antithrombotic treatment. The assumption that the risk of dying due to eICAD is probably higher than 1.4%, is supported by the 3-month death rate of 6.5% in a recent series of 290 dissection patients treated with endovascular recanalization or intravenous thrombolysis.^45^

For the outcome “death or disability,” the point estimate indicates that patients treated with anticoagulants might have twice the chance of avoiding death or disability compared with patients treated with antiplatelets. However, the confidence interval indicates that this effect could be much smaller (i.e. 50%) or larger (i.e. 160%).

For both co-primary outcomes, our key findings were predominantly driven by data from non-randomized studies, which provided >90% of the patients included. Therefore, the suggested benefit in favor of anticoagulants may have arisen from methodological biases. Antiplatelets could have been primarily applied in patients who were considered to have a poor prognosis, for example due to large infarcts^42,46^ or who were in a poor condition. Anticoagulation might be preferred in patients with a transient ischemic attack (TIA) or in patients presenting with pure local symptoms which have a better prognosis than those presenting with stroke.^47^

Regarding the secondary outcomes, there was an apparent trend in favor of anticoagulants for ischemic stroke. Interestingly, this effect was significant for the randomized trials, and missed significance for the non-randomized studies. Overall, the point estimate suggests a 50% higher chance to avoid ischemic stroke if patients were treated with anticoagulants than with antiplatelets, although the lower end of the confidence interval (0.88) included the possibility that antiplatelets might be superior. The potential benefit of anticoagulants might be explained by the assumption that emboli arising from the dissected artery may be of clinical importance and may cause fatal or disabling strokes.^48–52^

The results of our post-hoc sensitivity analyses showed that methodological heterogeneity had no substantial impact on our key findings, which might suggest that these findings are robust. More interestingly, the post-hoc sensitivity analyses comparing AC with AP stratified to the dichotomized length of follow-up indicated that any superiority of AC over AP (regarding death, disability, or ischemic stroke) might be present only in the earlier phase (within 3/6 months) but not later (i.e. ⩾1 year). However, we urge to a cautious interpretation of the aforementioned findings, considering that these analyses were performed post-hoc.

Regarding intra- or major extracranial hemorrhages, antiplatelets had an advantage over anticoagulants. Therefore, the observed benefit of anticoagulants in preventing ischemic stroke might come at the cost of an increase of bleeding complications. These observations resemble the key findings of the recently published STOP-CAD observational study, which reported that anticoagulation (compared to antiplatelets was associated with a non-significantly lower risk of subsequent ischemic stroke by day 30 (adjusted hazard ratio [HR], 0.71 [95% CI, 0.45–1.12]; *p* = 0.145) and by day 180 (adjusted HR, 0.80 [95% CI, 0.28–2.24]; *p* = 0.670), while anticoagulation therapy was not associated with a higher risk of major hemorrhage by day 30 (adjusted HR, 1.39 [95% CI, 0.35–5.45]; *p* = 0.637) but was by day 180 (adjusted HR, 5.56 [95% CI, 1.53–20.13]; *p* = 0.009).^53^

In this context, it is a challenge to understand the observed benefit of anticoagulants over antiplatelet regarding death and death or disability. We were not able to study, the impact of the occurrence of ischemic stroke versus bleeding complications on the co-primary outcomes of death and death or disability, respectively. One might speculate, that ischemic strokes – the complication for which anticoagulants seemed to be the more powerful preventive means – are the most frequent complication and/or have a bigger impact on functional outcome than the bleeding complications (for the prevention of which antiplatelets seemed preferable).

Our findings regarding ischemic stroke and major hemorrhages are in line with a recently published meta-analysis comparing AC with AP treatment of patients with cervical artery dissection – that is, ICAD combined with VAD.^10^ In this study, AC was superior to AP in reducing ischemic stroke but carried a higher major bleeding risk.^10^ Our research added that these findings seem applicable also to the group of patients with ICAD. More importantly and as a refinement, our analyses suggested that – in addition – AC might be superior to AP also in preventing death or disability.

We are aware of other important limitations. Outcome measurement was not applied uniformly in the included non-randomized studies which also differed in their focus. This included that some studies reported on “any ischemic stroke” while others only on “ipsilateral ischemic stroke.” It is therefore likely that there are important biases. Nonrandomized studies are known to be highly susceptible to bias and outcome events may be under-represented.^54^ Such biases encountered in the reported studies may be: reporting favorable cases; reports on selected cases as well as editorial biases, such as not allowing reports on already published issues by different authors; the choice of treatments may have been biased by the preference of the treating physicians.^55^ Moreover, the retrievable baseline data did not allow to stratify the analyses neither by type of presenting symptom nor by stroke severity in those patients presenting with stroke. This prevented us from studying whether imbalances in baseline variables might have influenced the seemingly better result for the anticoagulation group in avoiding death and death or disability.

The analyses across data from the two randomized trials were not sufficient to support or refute the suggested beneficial effect of anticoagulants because of the limited number of both participants and outcome events. Nevertheless, the point estimates of these analyses point in the same direction as those of the non-randomized studies, indicating that the observed superiority of the treatment with anticoagulants might not be caused by mere bias or chance.

In addition, we were not able to analyze the impact of direct oral anticoagulants or dual antiplatelets on our primary or secondary outcomes, because of the lack of analyzable data on this issue.^25^ This lack has also been reported in a recent meta-analysis.^10^ Comparisons of direct oral anticoagulants versus VKA or versus dual antiplatelets are of clinical interest and might be addressed by secondary analyses of the STOP-CAD-study.^53^

Furthermore, we focused on eICAD rather than on cervical artery dissections as it remains unclear whether carotid artery dissection and vertebral artery dissection can be regarded as one entity. However, given the known heterogeneity between ICAD and VAD patients, this approach – differing from several prior studies and meta-analyses –, may also be considered as advantage and novelty.

Finally, allocation of ICAD patients to antithrombotic treatment regimens has followed a universal approach in disregard of proven heterogeneity in patient-level baseline profiles and its meaning regarding response to specific antithrombotic treatment regimens.^9,56^

In conclusion, this systematic review suggests that the evidence considering antiplatelets as standard of care in eICAD is weak. Individualized treatment decisions balancing risks versus harms seem recommendable. In this context the co-primary outcomes death and death or disability, respectively might be of importance in shared decision making with patients.

## Supplemental Material

sj-jpg-1-eso-10.1177_23969873241292278 – Supplemental material for Antithrombotic drugs for carotid artery dissection: Updated systematic review Supplemental material, sj-jpg-1-eso-10.1177_23969873241292278 for Antithrombotic drugs for carotid artery dissection: Updated systematic review by Nikolaos S Avramiotis, Fabian Schaub, Sebastian Thilemann, Philippe Lyrer and Stefan T Engelter in European Stroke Journal

sj-jpg-2-eso-10.1177_23969873241292278 – Supplemental material for Antithrombotic drugs for carotid artery dissection: Updated systematic review Supplemental material, sj-jpg-2-eso-10.1177_23969873241292278 for Antithrombotic drugs for carotid artery dissection: Updated systematic review by Nikolaos S Avramiotis, Fabian Schaub, Sebastian Thilemann, Philippe Lyrer and Stefan T Engelter in European Stroke Journal

sj-jpg-3-eso-10.1177_23969873241292278 – Supplemental material for Antithrombotic drugs for carotid artery dissection: Updated systematic review Supplemental material, sj-jpg-3-eso-10.1177_23969873241292278 for Antithrombotic drugs for carotid artery dissection: Updated systematic review by Nikolaos S Avramiotis, Fabian Schaub, Sebastian Thilemann, Philippe Lyrer and Stefan T Engelter in European Stroke Journal

sj-jpg-4-eso-10.1177_23969873241292278 – Supplemental material for Antithrombotic drugs for carotid artery dissection: Updated systematic review Supplemental material, sj-jpg-4-eso-10.1177_23969873241292278 for Antithrombotic drugs for carotid artery dissection: Updated systematic review by Nikolaos S Avramiotis, Fabian Schaub, Sebastian Thilemann, Philippe Lyrer and Stefan T Engelter in European Stroke Journal

sj-jpg-5-eso-10.1177_23969873241292278 – Supplemental material for Antithrombotic drugs for carotid artery dissection: Updated systematic review Supplemental material, sj-jpg-5-eso-10.1177_23969873241292278 for Antithrombotic drugs for carotid artery dissection: Updated systematic review by Nikolaos S Avramiotis, Fabian Schaub, Sebastian Thilemann, Philippe Lyrer and Stefan T Engelter in European Stroke Journal

sj-jpg-6-eso-10.1177_23969873241292278 – Supplemental material for Antithrombotic drugs for carotid artery dissection: Updated systematic review Supplemental material, sj-jpg-6-eso-10.1177_23969873241292278 for Antithrombotic drugs for carotid artery dissection: Updated systematic review by Nikolaos S Avramiotis, Fabian Schaub, Sebastian Thilemann, Philippe Lyrer and Stefan T Engelter in European Stroke Journal

sj-jpg-7-eso-10.1177_23969873241292278 – Supplemental material for Antithrombotic drugs for carotid artery dissection: Updated systematic review Supplemental material, sj-jpg-7-eso-10.1177_23969873241292278 for Antithrombotic drugs for carotid artery dissection: Updated systematic review by Nikolaos S Avramiotis, Fabian Schaub, Sebastian Thilemann, Philippe Lyrer and Stefan T Engelter in European Stroke Journal

sj-pdf-8-eso-10.1177_23969873241292278 – Supplemental material for Antithrombotic drugs for carotid artery dissection: Updated systematic review Supplemental material, sj-pdf-8-eso-10.1177_23969873241292278 for Antithrombotic drugs for carotid artery dissection: Updated systematic review by Nikolaos S Avramiotis, Fabian Schaub, Sebastian Thilemann, Philippe Lyrer and Stefan T Engelter in European Stroke Journal

## References

[1] Nedeltchev K der Maur TA Georgiadis D , et al. Ischaemic stroke in young adults: predictors of outcome and recurrence. J Neurol Neurosurg Psychiatry 2005; 76: 191–195. 15654030 10.1136/jnnp.2004.040543 PMC1739502

[2] Anderson RM Schechter MM. A case of spontaneous dissecting aneurysm of the internal carotid artery. J Neurol Neurosurg Psychiatry 1959; 22: 195–201. 13793447 10.1136/jnnp.22.3.195 PMC497375

[3] Jentzer A. Dissecting aneurysm of the left internal carotid artery. Angiology 1954; 5: 232–234. 13158894 10.1177/000331975400500306

[4] Traenka C Dougoud D Simonetti BG , et al.; CADISP-Plus Study Group. Cervical artery dissection in patients ⩾60 years often painless, few mechanical triggers. Neurology 2017; 88: 1313–1320. 28258079 10.1212/WNL.0000000000003788

[5] Rosati LM Vezzetti A Redd KT , et al. Early anticoagulation or antiplatelet therapy is critical in craniocervical artery dissection: results from the COMPASS Registry. Cerebrovasc Dis 2020; 49: 369–374. 32731249 10.1159/000509415

[6] Markus HS Levi C King A , et al.; Cervical Artery Dissection in Stroke Study (CADISS) Investigators. Antiplatelet therapy vs anticoagulation therapy in cervical artery dissection: the cervical artery dissection in stroke study (CADISS) randomized clinical trial final results. JAMA Neurol 2019; 76: 657–664. 30801621 10.1001/jamaneurol.2019.0072 PMC6563567

[7] Engelter ST Traenka C Gensicke H , et al. Aspirin versus anticoagulation in cervical artery dissection (TREAT-CAD): an open-label, randomised, non-inferiority trial. Lancet Neurol 2021; 20: 341–350. 33765420 10.1016/S1474-4422(21)00044-2

[8] Debette S Mazighi M Bijlenga P , et al. ESO guideline for the management of extracranial and intracranial artery dissection. Eur Stroke J 2021; 6: XXXIX–LXXXVIII. 10.1177/23969873211046475 PMC8564160 34746432

[9] Kaufmann JE Harshfield EL Gensicke H , et al.; CADISS and TREAT-CAD Investigators. Antithrombotic treatment for cervical artery dissection: A systematic review and individual patient data meta-analysis. JAMA Neurol 2024; 81: 630–637. 38739383 10.1001/jamaneurol.2024.1141 PMC11091821

[10] Yaghi S Shu L Fletcher L , et al. Anticoagulation versus antiplatelets in spontaneous cervical artery dissection: a systematic review and meta-analysis. Stroke 2024; 55: 1776–1786. 38847098 10.1161/STROKEAHA.124.047310

[11] Markus HS Hayter E Levi C , et al.; CADISS Trial Investigators. Antiplatelet treatment compared with anticoagulation treatment for cervical artery dissection (CADISS): a randomised trial. Lancet Neurol 2015; 14: 361–367. 25684164 10.1016/S1474-4422(15)70018-9

[12] Debette S Leys D. Cervical-artery dissections: predisposing factors, diagnosis, and outcome. Lancet Neurol 2009; 8: 668–678. 19539238 10.1016/S1474-4422(09)70084-5

[13] Debette S Grond-Ginsbach C Bodenant M , et al.; Cervical Artery Dissection Ischemic Stroke Patients (CADISP) Group. Differential features of carotid and vertebral artery dissections: the CADISP study. Neurology 2011; 77: 1174–1181. 21900632 10.1212/WNL.0b013e31822f03fc

[14] Compter A Schilling S Vaineau CJ , et al.; For the CADISP-plus Consortium and CADISP-plus Consortium. Determinants and outcome of multiple and early recurrent cervical artery dissections. Neurology 2018; 91: e769–e780. 30068628 10.1212/WNL.0000000000006037

[15] Lee VH Brown Rd Jr Mandrekar JN , et al. Incidence and outcome of cervical artery dissection: a population-based study. Neurology 2006; 67: 1809–1812. 17130413 10.1212/01.wnl.0000244486.30455.71

[16] Keser Z Chiang CC Benson JC , et al. Cervical artery dissections: etiopathogenesis and management. Vasc Health Risk Manag 2022; 18: 685–700. 36082197 10.2147/VHRM.S362844 PMC9447449

[17] Arauz A Hoyos L Espinoza C , et al. Dissection of cervical arteries: long-term follow-up study of 130 consecutive cases. Cerebrovasc Dis 2006; 22: 150–154. 16691024 10.1159/000093244

[18] Caprio FZ Bernstein RA Alberts MJ , et al. Efficacy and safety of novel oral anticoagulants in patients with cervical artery dissections. Cerebrovasc Dis 2014; 38: 247–253. 25401389 10.1159/000366265

[19] Mustanoja S Metso TM Putaala J , et al. Helsinki experience on nonvitamin K oral anticoagulants for treating cervical artery dissection. Brain Behav 2015; 5: e00349. 26356074 10.1002/brb3.349 PMC4559015

[20] Lyrer P Engelter S. Antithrombotic drugs for carotid artery dissection. Cochrane Database Syst Rev 2003; (3): 14651858. DOI: 10.1002/14651858 12917890

[21] Page MJ McKenzie JE Bossuyt PM , et al. The PRISMA 2020 statement: an updated guideline for reporting systematic reviews. BMJ 2021; 372: n71. 10.1136/bmj.n71 PMC8005924 33782057

[22] Lyrer P Engelter S. Antithrombotic drugs for carotid artery dissection. Cochrane Database Syst Rev 2010; (10): CD000255. DOI: 10.1002/14651858.CD000255.pub2 PMC12365947 20927720

[23] Lyrer P Engelter S. Antithrombotic drugs for carotid artery dissection. Stroke 2004; 35: 613–614. 14739417 10.1161/01.STR.0000112970.63735.FC

[24] Traenka C Gensicke H Schaedelin S , et al.; TREAT-CAD Investigators. Biomarkers and antithrombotic treatment in cervical artery dissection - design of the TREAT-CAD randomised trial. Eur Stroke J 2020; 5: 309–319. 33072885 10.1177/2396987320921151 PMC7538765

[25] Essibayi MA Lanzino G Keser Z. Vitamin K antagonist versus novel oral anticoagulants for management of cervical artery dissection: interactive systematic review and meta-analysis. Eur Stroke J 2022; 7: 349–357. 36478754 10.1177/23969873221111051 PMC9720846

[26] Hatano S. Experience from a multicentre stroke register: a preliminary report. Bull World Health Organ 1976; 54: 541–553. 1088404 PMC2366492

[27] Higgins JP Altman DG Gøtzsche PC , et al.; Cochrane Bias Methods Group and Cochrane Statistical Methods Group. The Cochrane Collaboration's tool for assessing risk of bias in randomised trials. BMJ 2011; 343: d5928. 10.1136/bmj.d5928 PMC3196245 22008217

[28] Egger M Davey Smith G Schneider M , et al. Bias in meta-analysis detected by a simple, graphical test. BMJ 1997; 315: 629–634. 9310563 10.1136/bmj.315.7109.629 PMC2127453

[29] Antiplatelet Trialists’ Collaboration. Collaborative overview of randomised trials of antiplatelet therapy Prevention of death, myocardial infarction, and stroke by prolonged antiplatelet therapy in various categories of patients. BMJ 1994; 308: 81–106. DOI: 10.1136/bmj.308.6921.81 8298418 PMC2539220

[30] Higgins JPT Thompson SG . Quantifying heterogeneity in a meta-analysis. Stat Med 2002; 21: 1539–1558. 12111919 10.1002/sim.1186

[31] Traenka C Grond-Ginsbach C Goeggel Simonetti B , et al.; CADISP-Plus Study Group. Artery occlusion independently predicts unfavorable outcome in cervical artery dissection. Neurology 2020; 94: e170–e180. 10.1212/WNL.0000000000008654 PMC6988986 31757869

[32] Vineetha VS Sreedharan SE Sarma PS , et al. Antiplatelets versus anticoagulants in the treatment of extracranial carotid and vertebral artery dissection. Neurol India 2019; 67: 1056–1059. 31512634 10.4103/0028-3886.266290

[33] Kennedy F Lanfranconi S Hicks C , et al.; CADISS Investigators. Antiplatelets vs anticoagulation for dissection: CADISS nonrandomized arm and meta-analysis. Neurology 2012; 79: 686–689. 22855862 10.1212/WNL.0b013e318264e36b

[34] Sercl M Eichlova Z Barsa P , et al. Spontaneous dissection of internal carotid artery. Cesk Radiol 2020; 74: 131–138.

[35] Reges DS Mazzeo M Rosalino R , et al. Cervical arterial dissection: clinical characteristics in a neurology service in São Paulo, Brazil. Arq Neuropsiquiatr 2019; 77: 632–637. 31553393 10.1590/0004-282X20190108

[36] Brunser AM Lavados PM Hoppe A , et al. Transcranial Doppler as a predictor of ischemic events in carotid artery dissection. J Neuroimaging 2017; 27: 232–236. 27491878 10.1111/jon.12379

[37] Metso TM Metso AJ Salonen O , et al. Adult cervicocerebral artery dissection: a single-center study of 301 Finnish patients. Eur J Neurol 2009; 16: 656–661. 19220449 10.1111/j.1468-1331.2009.02535.x

[38] Georgiadis D Arnold M von Buedingen HC , et al. Aspirin vs anticoagulation in carotid artery dissection: a study of 298 patients. Neurology 2009; 72: 1810–1815. 19321846 10.1212/WNL.0b013e3181a2a50a

[39] Richaud J Lagarrigue J Lazorthes Y. [Traumatic injury affecting the extracranial portion of internal carotid artery (17 case reports) (author's transl)]. Neurochirurgie 1980; 26: 109–121. 7412977

[40] Saver J Easton J Hart R . Dissections and trauma of cervicocerebral arteries. In: H Barnett J Mohr B Stein , et al. (eds) Stroke. Pathophysiology, diagnosis and management. New York: Churchill Livingstone Inc, 1992, 2nd ed, pp. 671–681.

[41] Ast G Woimant F Georges B , et al. Spontaneous dissection of the internal carotid artery in 68 patients. Eur J Med 1993; 2: 466–472. 8258047

[42] Bogousslavsky J Despland PA Regli F. Spontaneous carotid dissection with acute stroke. Arch Neurol 1987; 44: 137–140. 3813930 10.1001/archneur.1987.00520140009010

[43] Colella JJ Diamond DL. Blunt carotid injury: reassessing the role of anticoagulation. Am Surg 1996; 62: 212–217. 8607581

[44] Wahl WL Brandt MM Thompson BG , et al. Antiplatelet therapy: an alternative to heparin for blunt carotid injury. Trauma 2002; 52: 896–901. 10.1097/00005373-200205000-00012 11988655

[45] Traenka C Lorscheider J Hametner C , et al.; For the EVA-TRISP Collaborators. Recanalization therapies for large vessel occlusion due to cervical artery dissection: a cohort study of the EVA-TRISP collaboration. J Stroke 2023; 25: 272–281. 37282374 10.5853/jos.2022.03370 PMC10250869

[46] Chen ST Ryu SJ Hsi MS. Cervico-cerebral artery dissection. Taiwan Yi Xue Hui Za Zhi 1984; 83: 846–861. 6596393

[47] Lichy C Metso A Pezzini A , et al.; Cervical Artery Dissection and Ischemic Stroke Patients-Study Group. Predictors of delayed stroke in patients with cervical artery dissection. Int J Stroke 2015; 10: 360–363. 23227939 10.1111/j.1747-4949.2012.00954.x

[48] Anson J Crowell RM. Cervicocranial arterial dissection. Neurosurg 1991; 29: 89–96. 10.1097/00006123-199107000-00015 1870693

[49] Droste DW Junker K Stögbauer F , et al. Clinically silent circulating microemboli in 20 patients with carotid or vertebral artery dissection. Cerebrovasc Dis 2001; 12: 181–185. 11641581 10.1159/000047701

[50] Koennecke HC Trocio Sh Jr Mast H , et al. Microemboli on transcranial Doppler in patients with spontaneous carotid artery dissection. J Neuroimaging 1997; 7: 217–220. 9344003 10.1111/jon199774217

[51] Oliveira V Batista P Soares F , et al. HITS in internal carotid dissections. Cerebrovasc Dis 2001; 11: 330–334. 11385213 10.1159/000047662

[52] Steinke W Schwartz A Hennerici M. Topography of cerebral infarction associated with carotid artery dissection. J Neurol 1996; 243: 323–328. 8965105 10.1007/BF00868406

[53] Yaghi S Shu L Mandel D , et al. Antithrombotic treatment for stroke prevention in cervical artery dissection: the STOP-CAD study. Stroke 2024; 55(4): 908–918. DOI: 10.1161/STROKEAHA.123.045731 38335240

[54] Chalmers TC Celano P Sacks HS , et al. Bias in treatment assignment in controlled clinical trials. N Engl J Med 1983; 309: 1358–1361. 6633598 10.1056/NEJM198312013092204

[55] Sackett DL. Bias in analytic research. J Chronic Dis 1979; 32: 51–63. 447779 10.1016/0021-9681(79)90012-2

[56] Kaufmann JE Gensicke H Schaedelin S , et al.; For the TREAT-CAD Trial. Toward individual treatment in cervical artery dissection: subgroup analysis of the TREAT-CAD randomized trial. Ann Neurol 2024; 95: 886–897. 38362818 10.1002/ana.26886

