# Supplementary figures and images for: Antithrombotic drugs for carotid artery dissection: Updated systematic review
Source: Eur Stroke J. 2024 Oct 26;10(2):339–49. doi: 10.1177/23969873241292278 (PMC11556626; doi:10.1177/23969873241292278)

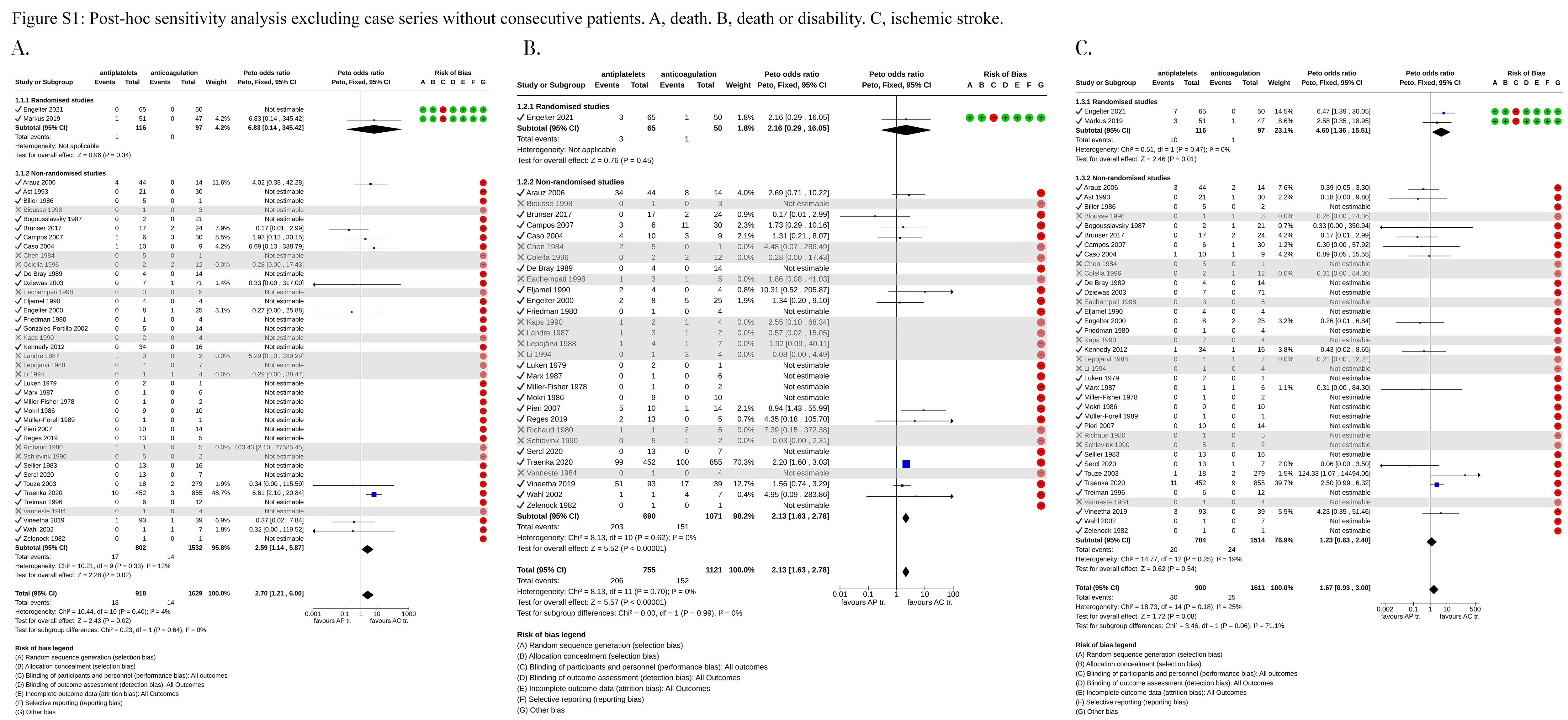

Supplement: sj-jpg-1-eso-10.1177_23969873241292278 – Supplemental material for Antithrombotic drugs for carotid artery dissection: Updated systematic review [file sj-jpg-1-eso-10.1177_23969873241292278.jpg]

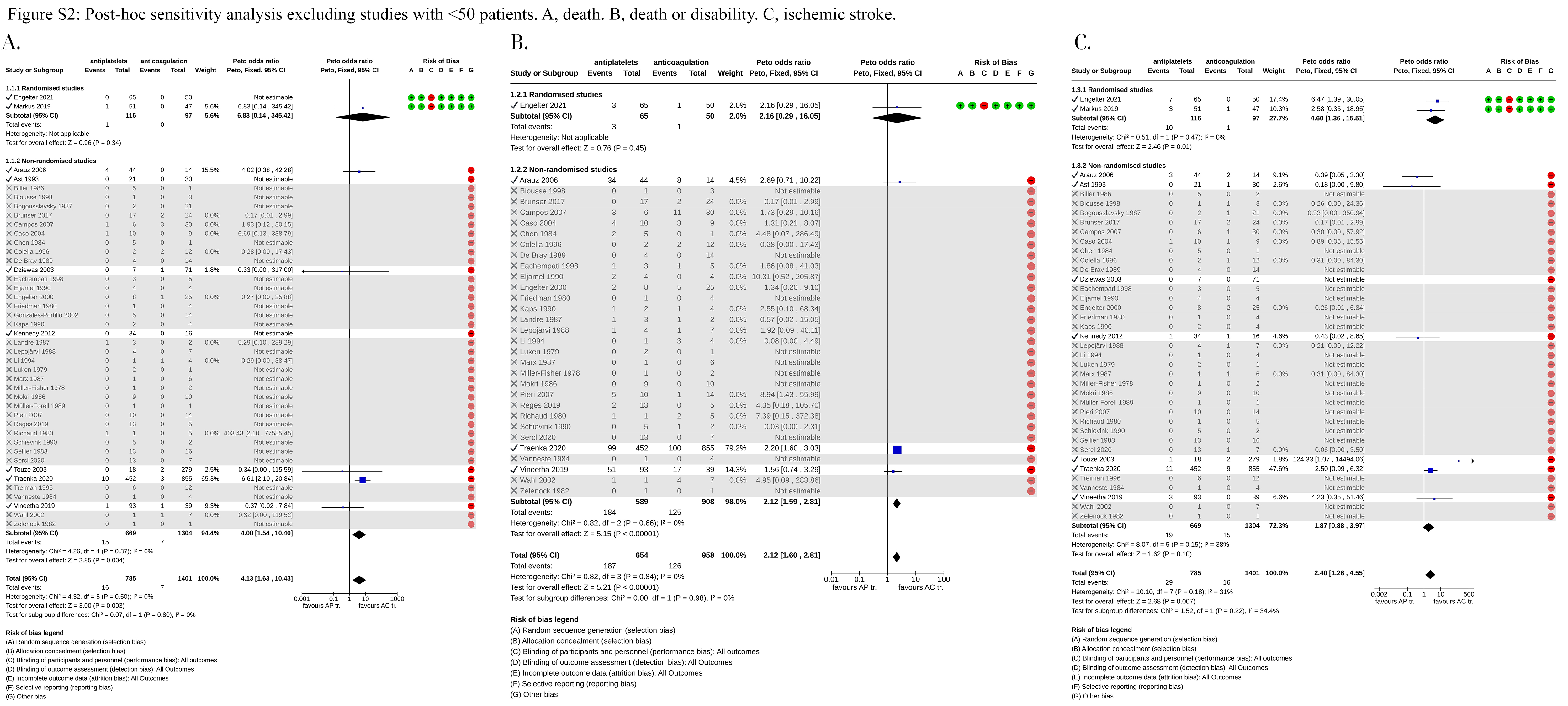

Supplement: sj-jpg-2-eso-10.1177_23969873241292278 – Supplemental material for Antithrombotic drugs for carotid artery dissection: Updated systematic review [file sj-jpg-2-eso-10.1177_23969873241292278.jpg]

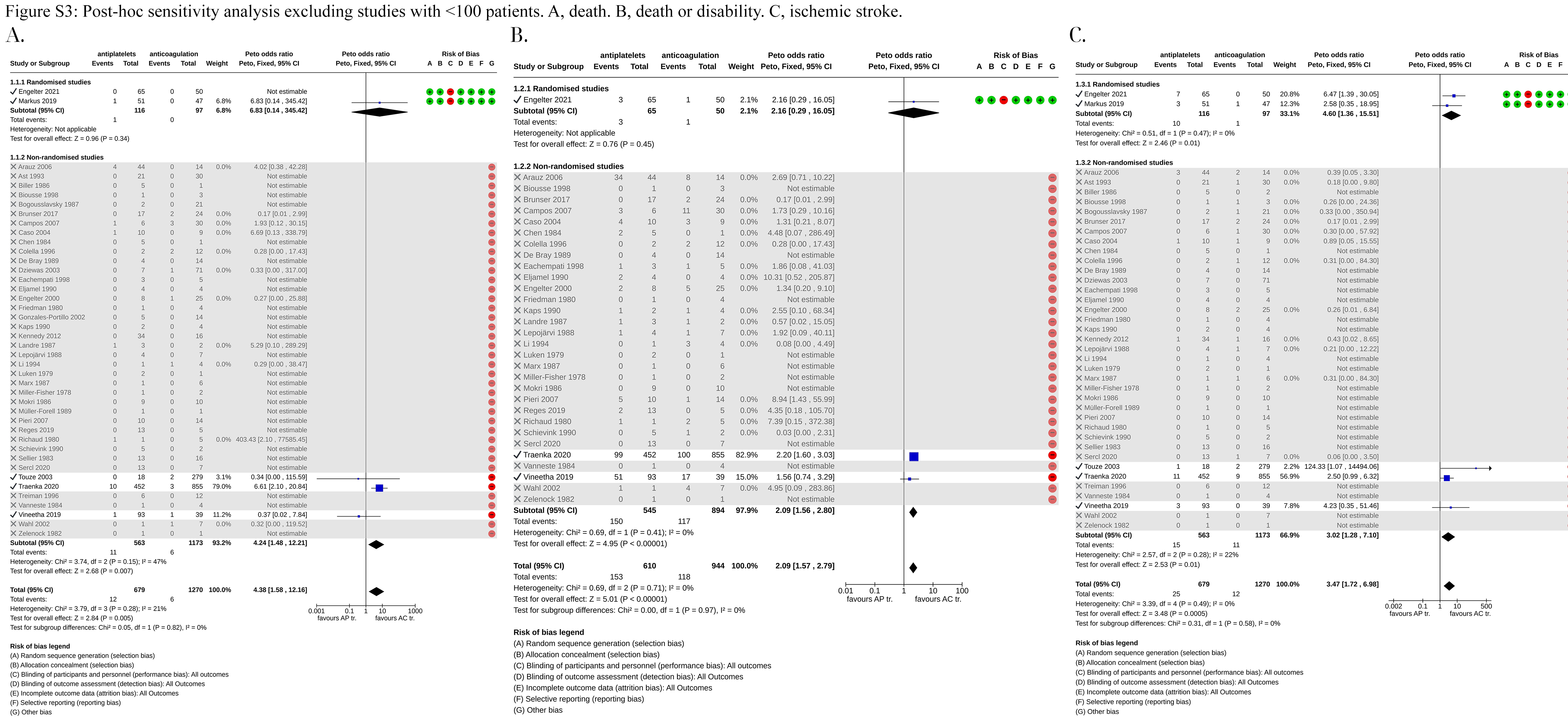

Supplement: sj-jpg-3-eso-10.1177_23969873241292278 – Supplemental material for Antithrombotic drugs for carotid artery dissection: Updated systematic review [file sj-jpg-3-eso-10.1177_23969873241292278.jpg]

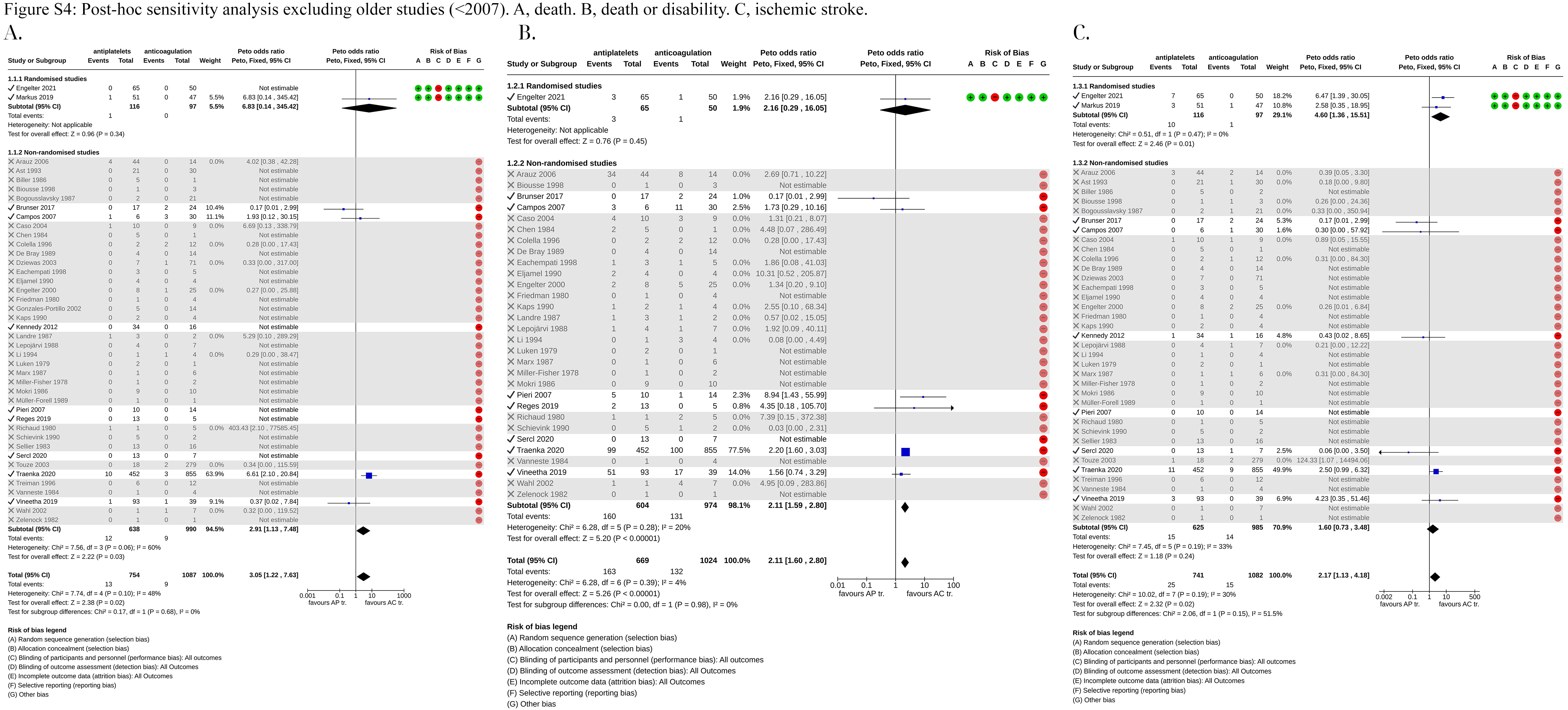

Supplement: sj-jpg-4-eso-10.1177_23969873241292278 – Supplemental material for Antithrombotic drugs for carotid artery dissection: Updated systematic review [file sj-jpg-4-eso-10.1177_23969873241292278.jpg]

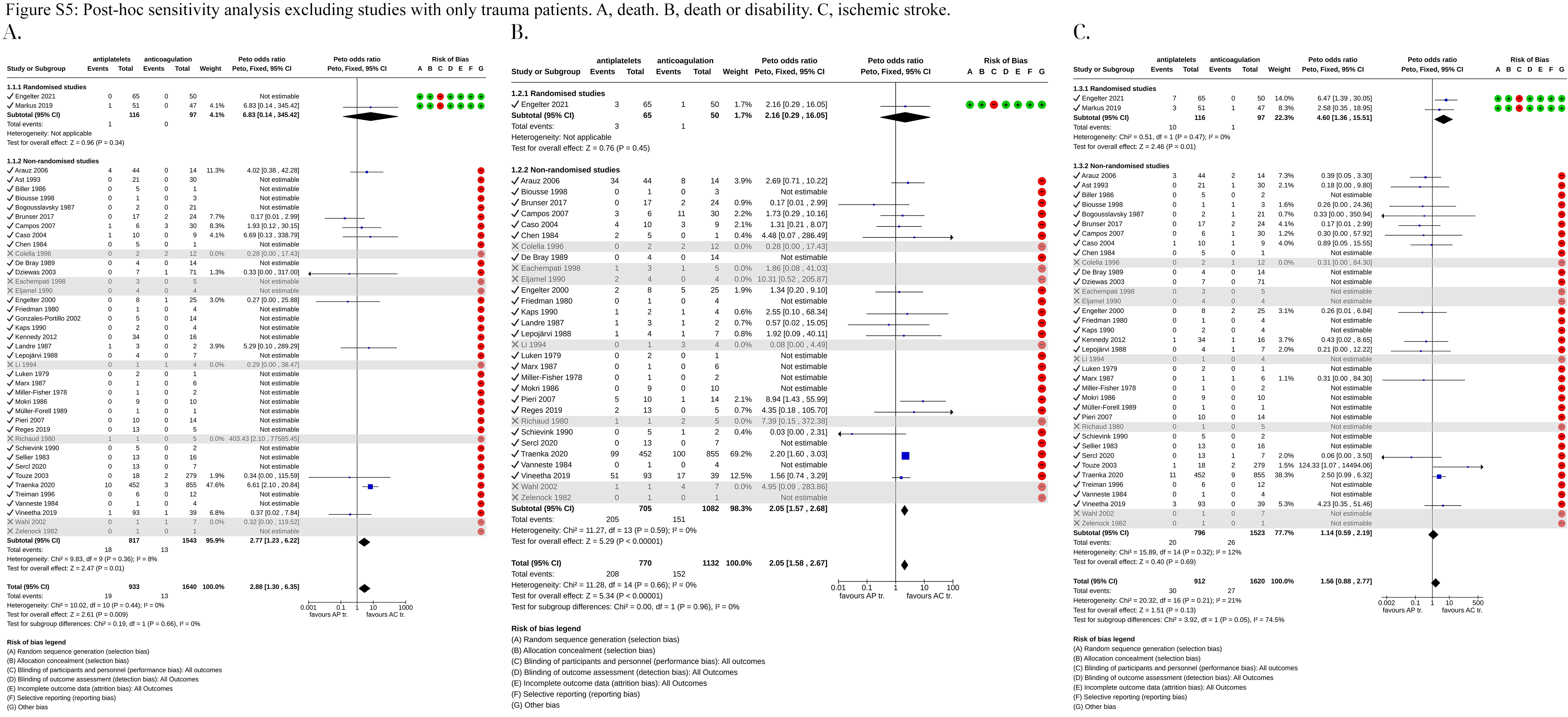

Supplement: sj-jpg-5-eso-10.1177_23969873241292278 – Supplemental material for Antithrombotic drugs for carotid artery dissection: Updated systematic review [file sj-jpg-5-eso-10.1177_23969873241292278.jpg]

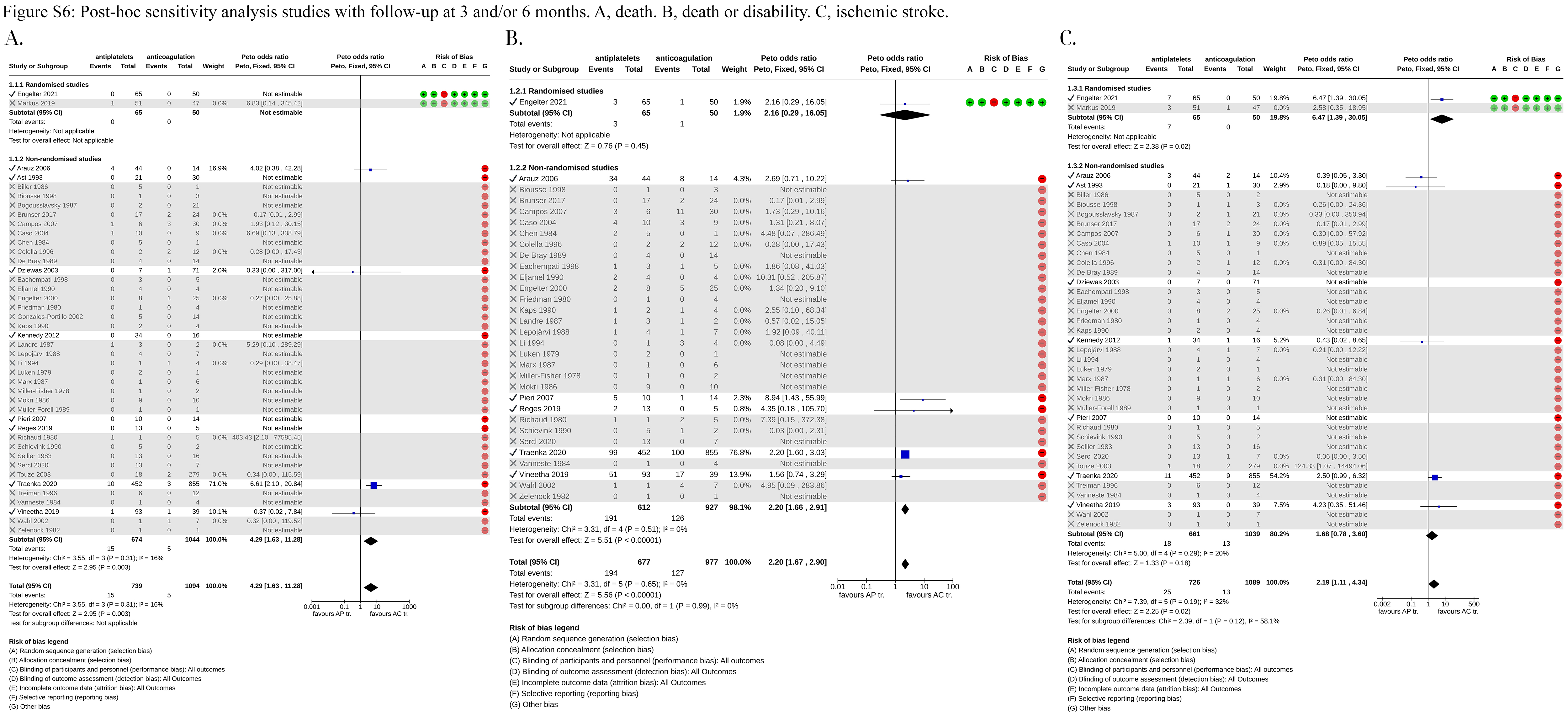

Supplement: sj-jpg-6-eso-10.1177_23969873241292278 – Supplemental material for Antithrombotic drugs for carotid artery dissection: Updated systematic review [file sj-jpg-6-eso-10.1177_23969873241292278.jpg]

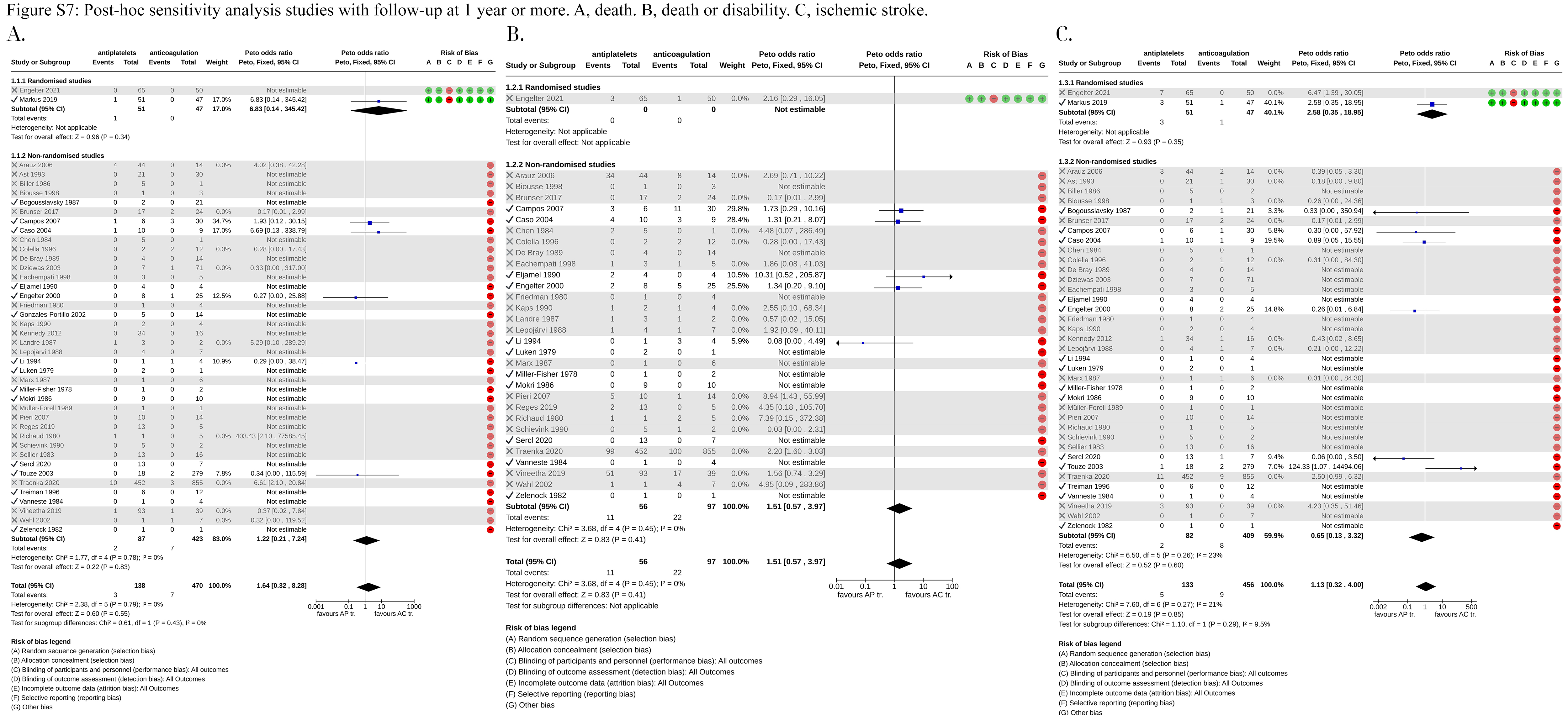

Supplement: sj-jpg-7-eso-10.1177_23969873241292278 – Supplemental material for Antithrombotic drugs for carotid artery dissection: Updated systematic review [file sj-jpg-7-eso-10.1177_23969873241292278.jpg]
