# Supplementary material for: Antithrombotic drugs for carotid artery dissection: Updated systematic review
Source: Eur Stroke J. 2024 Oct 26;10(2):339–49. doi: 10.1177/23969873241292278 (PMC11556626; doi:10.1177/23969873241292278)
Supplement: sj-pdf-8-eso-10.1177_23969873241292278 – Supplemental material for Antithrombotic drugs for carotid artery dissection: Updated systematic review [file sj-pdf-8-eso-10.1177_23969873241292278.pdf]

Table S1: Search Strategy CENTRAL

#1 MeSH descriptor: [Carotid Artery Injuries] explode all trees 20
#2 MeSH descriptor: [Carotid Artery, Internal, Dissection] this term only 5
#3 (carotid near/5 (injur* or dissection or trauma*)):ti,ab,kw 181
#4 MeSH descriptor: [Carotid Arteries] explode all trees 1122
#5 MeSH descriptor: [Carotid Artery Diseases] this term only 472
#6 MeSH descriptor: [Carotid Artery Thrombosis] this term only 18
#7 (carotid*):ti,ab,kw 8123
#8 {or #3-#7} 8123
#9 MeSH descriptor: [Aneurysm, Dissecting] explode all trees 98
#10 MeSH descriptor: [Aneurysm, False] this term only 26
#11 MeSH descriptor: [Aneurysm, Ruptured] explode all trees 196
#12 MeSH descriptor: [Wounds, Nonpenetrating] explode all trees 691
#13 (traumatic near/5 (dissection or aneurysm or pseudoaneurysm)):ti,ab,kw 15
#14 (blunt near/5 (injur* or trauma)):ti,ab,kw 497
#15 (dissecting aneurysm):ti,ab,kw 130
#16 MeSH descriptor: [Rupture, Spontaneous] this term only 109
#17 MeSH descriptor: [Rupture] this term only 565
#18 (spontaneous dissection):ti,ab,kw 71
#19 {or #9-#18} 2149
#20 #8 and #19 70
#21 #1 or #2 or #20 83
#22 MeSH descriptor: [Anticoagulants] explode all trees 4578
#23 MeSH descriptor: [Platelet Aggregation Inhibitors] explode all trees 3850
#24 MeSH descriptor: [Blood Platelets] this term only and with qualifier(s): [drug effects - DE] 1103
#25 MeSH descriptor: [Platelet Aggregation] this term only and with qualifier(s): [drug effects - DE] 1447
#26 MeSH descriptor: [Fibrinolytic Agents] explode all trees 2185
#27 MeSH descriptor: [Thrombolytic Therapy] this term only 1625
#28 MeSH descriptor: [Thromboembolism] this term only and with qualifier(s): [drug therapy - DT] 138
#29 MeSH descriptor: [Thrombosis] this term only and with qualifier(s): [drug therapy - DT] 205
#30 (antiplatelet* or antithromb* or anticoag* or ((thromb* or blood clot* or platelet aggregat* or factor Xa or factor X* or factor 10* or factor II* or factor 2*) near/5 (block* or antagon* or inhibitor*)) or DOAC or NOAC or rivaroxaban or apixaban or edoxaban or dabigatran):ti,ab,kw 61044
#31 (Vitamin K antagonist* or VKA*):ti,ab,kw 1114
#32 (aspirin* or acetyl?salicylic acid* or abciximab or clopidogrel or dipyridamole or eptifibatide or prasugrel or ticagrelor or ticlopidine or tirofiban):ti,ab,kw 20859
#33 (heparin* or LMWH or coumarin* or coumadin* or warfarin or phenprocoumon or acenocoumarol or dalteparin or enoxaparin or tinzaparin or certoparin or nadroparin or reviparin or fondaparinux or argatroban or danaparoid or bivalirudin or desirudin or lepirudin or efegatran or inogatran or melagatran or ximelagatran):ti,ab,kw 18409
#34 {or #22-#33} 82750
#35 #21 and #34 15

For Peer Review

Table S2: Search strategy for MEDLINE (Ovid).

1. exp carotid artery injuries/ or carotid artery, internal, dissection/
2. (carotid adj5 (injur$ or dissection or trauma$)).tw.
3. exp carotid arteries/
4. carotid artery diseases/
5. carotid artery thrombosis/
6. carotid$.tw.
7. 3 or 4 or 5 or 6
8. exp aneurysm, dissecting/ or aneurysm, false/ or exp aneurysm, ruptured/
9. exp wounds, nonpenetrating/
10. (traumatic adj5 (dissection or aneurysm or pseudoaneurysm)).tw.
11. (blunt adj5 (injur$ or trauma)).tw.
12. dissecting aneurysm.tw.
13. rupture, spontaneous/ or rupture/
14. spontaneous dissection.tw.
15. 8 or 9 or 10 or 11 or 12 or 13 or 14
16. 7 and 15
17. 1 or 2 or 16
18. exp anticoagulants/ or exp platelet aggregation inhibitors/
19. Blood Platelets/de [Drug Effects]
20. Platelet Aggregation/de [Drug Effects]
21. exp Fibrinolytic Agents/
22. Thrombolytic Therapy/
23. Thromboembolism/dt [Drug Therapy]
24. Thrombosis/dt [Drug Therapy]
25. (antiplatelet$ or antithromb$ or anticoag$ or ((thromb$ or blood clot$ or platelet aggregat$ or factor Xa or factor X$ or factor 10$ or factor II$ or factor 2$) adj5 (block$ or antagon$ or inhibitor$)) or DOAC or NOAC or rivaroxaban or apixaban or edoxaban or dabigatran).tw.
26. (Vitamin K antagonist$ or VKA$).tw.
27. (aspirin$ or acetyl?salicylic acid$ or abciximab or clopidogrel or dipyridamole or eptifibatide or prasugrel or ticagrelor or ticlopidine or tirofiban).tw.
28. (heparin$ or LMWH or coumarin$ or coumadin$ or warfarin or phenprocoumon or acenocoumarol or dalteparin or enoxaparin or tinzaparin or certoparin or nadroparin or reviparin or fondaparinux or argatroban or danaparoid or bivalirudin or desirudin or lepirudin or efegatran or inogatran or melagatran or ximelagatran).tw.
29. or/18-28
30. randomized controlled trial.pt.
31. controlled clinical trial.pt.
32. randomized.ab.
33. placebo.ab.
34. drug therapy.fs.
35. randomly.ab.

For Peer Review

36. trial.ti.
37. groups.ab.
38. or/30-37
39. exp animals/ not humans.sh.
40. 38 not 39
41. 17 and 29 and 40

Table S3: Search strategy for EMBASE (Ovid).

1. carotid artery injury/
2. exp carotid artery aneurysm/
3. (carotid adj5 (injur$ or dissection or trauma$)).tw.
4. exp carotid artery/
5. carotid artery disease/ or exp carotid artery anomaly/ or carotid artery bruit/ or carotid artery calcification/ or exp carotid artery obstruction/ or carotid atherosclerosis/
6. carotid$.tw.
7. 4 or 5 or 6
8. aneurysm/ or exp aneurysm rupture/ or dissecting aneurysm/ or exp intracranial aneurysm/
9. exp aneurysm rupture/
10. blunt trauma/
11. (traumatic adj5 (dissection or aneurysm or pseudoaneurysm)).tw.
12. (blunt adj5 (injur$ or trauma)).tw.
13. dissecting aneurysm.tw.
14. rupture/
15. spontaneous dissection.tw.
16. or/8-15
17. 7 and 16
18. 1 or 2 or 17
19. exp anticoagulant agent/ or anticoagulant therapy/
20. thrombocyte/
21. thrombocyte aggregation/
22. exp fibrinolytic agent/
23. exp fibrinolytic therapy/
24. exp thromboembolism/dt [Drug Therapy]
25. (antiplatelet$ or antithromb$ or anticoag$ or ((thromb$ or blood clot$ or platelet aggregat$ or factor Xa or factor X$ or factor 10$ or factor II$ or factor 2$) adj5 (block$ or antagon$ or inhibitor$)) or DOAC or NOAC or rivaroxaban or apixaban or edoxaban or dabigatran).tw.
26. (Vitamin K antagonist$ or VKA$).tw.
27. (aspirin$ or acetyl?salicylic acid$ or abciximab or clopidogrel or dipyridamole or eptifibatide or ketanserin or prasugrel or ticagrelor or ticlopidine or tirofiban).tw.
28. (heparin$ or LMWH or coumarin$ or coumadin$ or warfarin or phenprocoumon or acenocoumarol or dalteparin or enoxaparin or tinzaparin or certoparin or nadroparin or reviparin or fondaparinux or argatroban or danaparoid or bivalirudin or desirudin or lepirudin or efegatran or inogatran or melagatran or ximelagatran).tw.
29. or/19-28

For Peer Review

30. Randomized Controlled Trial/ or "randomized controlled trial (topic)"/
31. Randomization/
32. Controlled clinical trial/ or "controlled clinical trial (topic)"/
33. control group/ or controlled study/
34. clinical trial/ or "clinical trial (topic)"/ or phase 1 clinical trial/ or phase 2 clinical trial/ or phase 3 clinical trial/ or phase 4 clinical trial/
35. Crossover Procedure/
36. Double Blind Procedure/
37. Single Blind Procedure/ or triple blind procedure/
38. placebo/ or placebo effect/
39. (random$ or RCT or RCTs).tw.
40. (controlled adj5 (trial$ or stud$)).tw.
41. (clinical$ adj5 trial$).tw.
42. ((control or treatment or experiment$ or intervention) adj5 (group$ or subject$ or patient$)).tw.
43. (quasi-random$ or quasi random$ or pseudo-random$ or pseudo random$).tw.
44. ((control or experiment$ or conservative) adj5 (treatment or therapy or procedure or manage$)).tw.
45. ((singl$ or doubl$ or tripl$ or trebl$) adj5 (blind$ or mask$)).tw.
46. (cross-over or cross over or crossover).tw.
47. (placebo$ or sham).tw.
48. trial.ti.
49. (assign$ or allocat$).tw.
50. controls.tw.
51. or/30-50
52. (exp animal/ or animal.hw. or nonhuman/) not (exp human/ or human cell/ or (human or humans).ti.)
53. 51 not 52
54. 18 and 29 and 53

For Peer Review

Table S4: GRADE

Antiplatelets compared to anticoagulation for carotid artery dissection

Patient or population: carotid artery dissection

Setting:

Intervention: antiplatelets

Comparison: anticoagulation

Outcomes	Anticipated absolute effects* (95% CI)		Relative effect (95% CI)	№ of participants (studies)	Certainty of the evidence (GRADE)	Comments
	**Risk with anticoagulation**	**Risk with antiplatelets**				
Death from all causes - Randomized studies	0 per 1000	**0 per 1000** (0 to 0)	**OR 6.83** (0.14 to 345.42)	213 (2 RCTs)	⊕⊕◯◯ Low[a]	
Death from all causes - Non-randomized studies	11 per 1000	**27 per 1000** (13 to 57)	**OR 2.6** (1.2 to 5.6)	2411 (40 non-randomized studies)	⊕◯◯◯ Very low[b,c,d]	
Death from all causes - total	10 per 1000	**27 per 1000** (13 to 55)	**OR 2.69** (1.27 to 5.72)	2624 (42 non-randozised studies)	⊕◯◯◯ Very low[b,c]	Randomized and Non-Randomized Trials.
Death or disability - Randomized studies	20 per 1000	**42 per 1000** (6 to 247)	**OR 2.16** (0.29 to 16.05)	115 (1 RCT)	⊕⊕◯◯ Low[a]	
Death or disability - Non-randomized studies	146 per 1000	**259 per 1000** (212 to 312)	**OR 2.05** (1.58 to 2.66)	1838 (30 non-randomized studies)	⊕◯◯◯ Very low[b,c]	
Death or disability - total	140 per 1000	**250 per 1000** (205 to 302)	**OR 2.05** (1.58 to 2.66)	1953 (31 non-randomized studies)	⊕◯◯◯ Very low[b,c]	Randomized and Non-Randomized Trials.
Ischemic stroke (during follow-up) - Randomized studies	10 per 1000	**46 per 1000** (14 to 139)	**OR 4.60** (1.36 to 15.51)	213 (2 RCTs)	⊕⊕⊕◯ Moderate[e]	
Ischemic stroke (during follow-up) - Non-randomized studies	17 per 1000	**19 per 1000** (10 to 36)	**OR 1.12** (0.59 to 2.14)	2370 (37 non-randomized studies)	⊕◯◯◯ Very low[b,c,d,f]	
Ischemic stroke (during follow-up) - total	17 per 1000	**26 per 1000** (15 to 44)	**OR 1.53** (0.86 to 2.71)	2583 (39 non-randomized studies)	⊕◯◯◯ Very low[b,c,d,f]	Randomized and Non-Randomized Trials.

For Peer Review

Antiplatelets compared to anticoagulation for carotid artery dissection

Patient or population: carotid artery dissection

Setting:

Intervention: antiplatelets

Comparison: anticoagulation

Outcomes	**Anticipated absolute effects*** (95% CI)		Relative effect (95% CI)	№ of participants (studies)	Certainty of the evidence (GRADE)	Comments
	**Risk with anticoagulation**	**Risk with antiplatelets**				
Symptomatic intracranial hemorrhage - Randomized studies	Not pooled	Not pooled	Not pooled	(2 RCTs)	-	No outcome events detected.
Symptomatic intracranial hemorrhage - Non-randomized studies	15 per 1000	**4 per 1000** (1 to 13)	**OR 0.25** (0.07 to 0.86)	1128 (29 non-randomized studies)	⊕◯◯◯ Very low[b,c]	
Symptomatic intracranial hemorrhage - total	14 per 1000	**3 per 1000** (1 to 12)	**OR 0.25** (0.07 to 0.86)	1341 (31 non-randomized studies)	⊕◯◯◯ Very low[b,c,e]	Randomized and Non-Randomized Trials.
Major extracranial hemorrhage - Randomized studies	10 per 1000	**1 per 1000** (0 to 52)	**OR 0.10** (0.00 to 5.23)	213 (2 RCTs)	⊕⊕◯◯ Low[a]	
Major extracranial hemorrhage - Non-randomized studies	14 per 1000	**3 per 1000** (0 to 20)	**OR 0.19** (0.02 to 1.48)	865 (16 non-randomized studies)	⊕◯◯◯ Very low[b,c,g]	
Major extracranial hemorrhage - total	13 per 1000	**2 per 1000** (0 to 14)	**OR 0.17** (0.03 to 1.03)	1078 (18 non-randomized studies)	⊕◯◯◯ Very low[b,c,g]	Randomized and Non-Randomized Trials.

***The risk in the intervention group** (and its 95% confidence interval) is based on the assumed risk in the comparison group and the **relative effect** of the intervention (and its 95% CI).

CI: confidence interval; **OR:** odds ratio

GRADE Working Group grades of evidence
High certainty: we are very confident that the true effect lies close to that of the estimate of the effect.
Moderate certainty: we are moderately confident in the effect estimate: the true effect is likely to be close to the estimate of the effect, but there is a possibility that it is substantially different.
Low certainty: our confidence in the effect estimate is limited: the true effect may be substantially different from the estimate of the effect.
Very low certainty: we have very little confidence in the effect estimate: the true effect is likely to be substantially different from the estimate of effect.

For Peer Review

Explanations

[a]Small number of patients and events. The 95% CI is very large overlapping with no- and significant effect.

[b]Serious risk of bias of the whole body of evidence of non-randomized trials.

[c]Patient population of NRCTs very variable.

[d]Funnel Plot for non-randomized trials skewed in favor of Antiplatelets.

[e]Small number of patients and events. The 95% CI does not include the no-effect.

[f]>2000 Patients, 95% CI include no-effect.

[g]Small information size but not wide 95% CI

For Peer Review

Table S5: Characteristics of included studies with risk of bias assessment.

Study – Year	Data source	Methods	Participants	Interventions	Outcomes	Notes	Risk of Bias	
							**Judgement**	**Support**
Arauz 2006	PUB	Observational study, single-center, stroke registry based, traumatic and spontaneous dissections	58 eICAD stroke patients (among 130 patients with cervical artery dissection), mixed etiology	13 anticoagulation, 45 antiplatelets	Death, stroke during follow up (recurrent), modified Rankin scale at 6 months, recanalisation, intracranial or systemic bleeding	Mexico	High risk	Serious risk of bias (Confounding, Selection, Information, Reporting)
Ast 1993	PUB	Observational study, multicenter, retrospective from medical records	68 patients with eICAD, 21 with stroke, 15 with TIA or AF. Spontanneous.	30 anticoagulants, 21 antiplatelet drugs, 9 'no treatment', 2 surgery, 6 not mentioned	Death, stroke (ischemic, hemorrhagic, side not specified), TIA. Follow-up at 3 months.	France	High risk	Serious risk of bias (Confounding, Selection, Information, Reporting)
Biller 1986	PUB	Observational study, retrospective from angiographical database	11 patients with eICAD, 10 with stroke, 1 with Horner's syndrome. Mixed etiology.	2 anticoagulants, 5 antiplatelet drugs, 4 surgery	Death, complications including stroke (ischemic, hemorrhagic, side not specified)	USA	High risk	Serious risk of bias (Confounding, Selection, Information, Reporting)

For Peer Review

Biousse 1998	PUB	Observational study, selection from large series of eICAD	4 patients with eICAD, 4 with ocular ischaemia. Mixed etiology.	3 anticoagulants, 1 antiplatelet drugs	Death, disability, stroke (side not specified), symptomatic intracranial hemorrhage	France	High risk	Serious risk of bias (Confounding, Selection, Information, Reporting)
Bogousslavsky 1987	PUB	Observational study, data from Lausanne Stroke Registry, consecutive patients with first-ever stroke	30 patients with eICAD, 12 with stroke, 5 TIA or AF, 2 monocular blindness, 11 headache. Mixed etiology.	21 anticoagulants, 2 antiplatelet drugs, 7 'no treatment' (these patients died)	Death, stroke (ipsilateral), Reopening at 6 months, Follow-up >1 year.	Switzerland	High risk	Serious risk of bias (Confounding, Selection, Information, Reporting)
Brunser 2017	MIX	Observational Study	41 patients with 45 eICAD and 11 eVAD. 0 with Stroke, 9 with TIA, 26 with pain, 22 with Horner's. Spontaneous.	24 anticoagulants, 17 antiplatelets	Death, disability, stroke (ipsilateral), TIA, sICH, major extracranial hemorrhage	Chile, Additional information obtained through personal communication	High risk	Serious risk of bias (Confounding, Selection, Information, Reporting)
Campos 2007	MIX	Observational study	60 patient with cervical artery dissection, among those 36 with eICAD; all but 2 patients had ischemic events before	6 eICAD patients had anticoagulation, 30 had antiplatelets	Death, disability, stroke (ipsilateral), bleeding complications. Follow-up >1 year.	Sao Paulo, Brazil, Additional information obtained by personal communication	High risk	Serious risk of bias (Confounding, Selection, Information, Reporting)

For Peer Review

			diagnosis of dissection. Spontaneous.					
Caso 2004	MIX	Observational study, single center, consecutive patients	19 eICAD with stroke or TIA (all had visible infarcts), only spontaneous eICAD	9 anticoagulants, 10 antiplatelets	Death, disability, stroke (side not specified), recanalisation, symptomatic intracranial hemorrhage, major extracranial hemorrhage. Follow-up >1 year.	Italy, additional information obtained by personal communication, 1 patient with anticoagulation suffered a retinal infarction	High risk	Serious risk of bias (Confounding, Selection, Information, Reporting)
Chen 1984	PUB	Observational study, selection not reported	7 patients with eICAD	1 anticoagulants, 5 antiplatelet drugs, 1 surgery (EC/IC bypass)	Death, disability, stroke (side unavailable) (ischemic, hemorrhagic)	Taiwan	High risk	Serious risk of bias (Confounding, Selection, Information, Reporting)
Colella 1996	PUB	Observational study, case series from motor vehicle accidents or other trauma (2 falls)	18 patients with eICAD. Traumatic.	12 anticoagulants, 2 antiplatelet drugs, 2 no treatment, 2 surgery (type of intervention not stated)	Death, disability, stroke (side not specified) (ischemic, hemorrhagic),	USA, Confounding with primary trauma possible	High risk	Serious risk of bias (Confounding, Selection, Information, Reporting)
De Bray 1989	PUB	Observational study for ultrasound	20 patients with eICAD, 12 with stroke, 6 with TIA,	12 anticoagulants, 4 antiplatelet drugs, 2 no	Death, disability, stroke (side not specified), bleeding complications,	France,	High risk	Serious risk of bias (Confounding,

For Peer Review

		diagnosis	referred for ultrasound. Spontaneous.	treatment, 2 not mentioned				Selection, Informatio n, Reporting)
Dziewas 2003	MIX	Observatio nal study of cervical artery dissection	78 patients with eICAD, 55 with stroke, 10 with TIA, 13 with only local signs. Mixed etiology.	7 antiplatelets, 71 anticoagulation	Death, stroke (side not specified), symptomatic intracranial hemorrhage, major extracranial hemorrhage, Follow up 6 months	Munster, Germany, Additional information obtained by personal communication	High risk	Serious risk of bias (Confound ing, Selection, Informatio n, Reporting)
Eachempati 1998	PUB	Observatio nal study, case series of trauma registry	11 patients with traumatic eICAD	2 no treatment, 3 antiplatelet drugs, 5 anticoagulants, 1 surgery	Death, disability, bleeding complication. Stroke not reported.	USA, Confounding with primary trauma injuries possible	High risk	Serious risk of bias (Confound ing, Selection, Informatio n, Reporting)
Eljamel 1990	PUB	Observatio nal study for ultrasound diagnosis	8 patients with eICAD, 5 stroke, 3 TIA. Traumatic.	4 anticoagulants, 4 antiplatelet drugs	Death, disability, stroke (any), intracranial bleed. Follow-up >1 year.	UK	High risk	Serious risk of bias (Confound ing, Selection, Informatio n, Reporting)
Engelter 2000	PUB	Observatio nal, retrospecti ve study on long-term outcome	33 patients with eICAD: 20 stroke, 6 TIA, 7 non-ischemic. Mixed etiology.	25 anticoagulants, 8 antiplatelets	Death, disability, stroke (side not specified), TIA, any hemorrhage, seizures. Follow-up >1 year.	Basel, Switzerland	High risk	Serious risk of bias (Confound ing, Selection, Informatio n,

For Peer Review

								Reporting)
Engelter 2021	MIX	multicenter , randomized, open-label, non-inferiority trial with blinded assessment of outcome events	115 patients with eICAD were included in the per-protocol analysis. Mixed etiology.	65 patients received aspirin as antiplatelet therapy and 50 received vitamin K antagonist as anticoagulant	Ischemic stroke (ipsilateral), major extracranial hemorrhage, symptomatic intracranial hemorrhage, death, disability clinical outcomes were assessed at 90 days after treatment initiation (3 months).	Switzerland, Germany, Denmark1 ischemic stroke in the AP group is retinal infarction. Additional information obtained through personal communication	Table S6	
Friedman 1980	PUB	Observational study, consecutive patients	12 patients with eICAD, symptoms not mentioned. Mixed etiology.	4 anticoagulants, 1 antiplatelets, 5 no treatment, 2 surgery (1 trapping, 1 EC/IC bypass)	Death, disability, stroke (ipsilateral), symptomatic intracranial hemorrhage,	USA	High risk	Serious risk of bias (Confounding, Selection, Information, Reporting)
Georgiadis 2009	PUB	Observational study, databank-based, consecutive patients	298 patients with eICAD Presenting symptoms: ischemic stroke 118/202 with anticoagulants and 47/96 with antiplatelets; TIA 25/202 with	202 anticoagulants, 96 antiplatelet agents alone Those with surgical/endovascular treatments were excluded (17) as were those on aspirin followed by	Death, ischemic stroke (ipsilateral), TIA, symptomatic intracranial hemorrhage, major extracranial hemorrhage (assessment at 3 months)	Switzerland (Zurich, Bern) Data overlap with Traenka 2020 for the outcomes of death and ischemic stroke	High risk	Serious risk of bias (Confounding, Selection, Information, Reporting)

For Peer Review

			anticoagulants and 12/96 with antiplatelets, AF 7/202 with anticoagulants and 1/96 with antiplatelets, pure local signs 45/202 with anticoagulants and 35/96 with antiplatelets; asymptomatic 7/202 with anticoagulants and 1/95 with antiplatelets. Spontaneous.	warfarin (30), those with no antithrombotic treatment and those who died within 7 days due to malignant MCA infarction (8, no treatment modalities mentioned)				
Gonzales-Portillo 2002	PUB	Observational study, databank-based, consecutive patients	27 patients with cervical artery dissection (22 spontaneous, 5 traumatic): 21 strokes, 3 TIA, 3 non-ischemic symptoms), 19 patients had eICAD (2 bilateral), 8 had eVAD. Mixed etiology).	5 eICAD patients had antiplatelets, 14 had anticoagulants	Death, (strokes mentioned, but not stratified to treatment, side not specified), mean follow up 58 months	Indiana, USA	High risk	Serious risk of bias (Confounding, Selection, Information, Reporting)

For Peer Review

Kaps 1990	PUB	Observational study for ultrasound diagnosis, case series	11 patients with eICAD, 2 with stroke, 3 with TIA. Spontaneous.	4 anticoagulants, 2 antiplatelet drugs, 2 no treatment	Death, disability, stroke (ischemic, hemorrhagic, side not specified), follow-up at 6 months.	Germany	High risk	Serious risk of bias (Confounding, Selection, Information, Reporting)
Kennedy 2012	PUB	Multicenter prospective, non-randomized study	50 Patients with eICAD. Mixed etiology.	16 patients anticoagulants, 34 patients antiplatelets	Death, TIA, stroke (ipsilateral). Follow-up at 3 months	UK, Australia	High risk	Serious risk of bias (Confounding, Selection, Information, Reporting)
Landre 1987	PUB	Observational study, case series	5 patients with eICAD, 5 with stroke. Spontaneous.	2 anticoagulants, 3 antiplatelet drugs	Death, disability	France	High risk	Serious risk of bias (Confounding, Selection, Information, Reporting)
Lepojärvi 1988	PUB	Observational study, selection not reported	13 patients with eICAD, 8 with stroke, 3 with TIA. Spontaneous.	7 anticoagulants, 4 antiplatelet drugs, 1 no treatment, 1 surgery (ligation)	Death, disability, stroke (side not specified)	Finland	High risk	Serious risk of bias (Confounding, Selection, Information, Reporting)
Li 1994	PUB	Observational study, case	7 patients with eICAD, 7 with stroke.	4 anticoagulants, 1 antiplatelet	Death, disability, stroke (side not specified), TIA. Follow-up >1 year.	Canada, 2 deaths due to suicide,	High risk	Serious risk of bias (Confound

For Peer Review

		series from trauma patients	Traumatic.	drugs, 2 surgery (1 embolectomy, 1 endarterectomy)		confounding with trauma and premorbid status possible		ing, Selection, Informatio n, Reporting)
Luken 1979	PUB	Observatio nal study, consecutiv e case series	10 patients with eICAD, 4 with TIA or AF, 9 with pain. Spontaneous.	1 anticoagulants, 2 antiplatelet drugs, 3 no treatment, 4 surgery (2 ligations, 1 silverstone clamp, 1 embolectomy)	Death, disability, stroke (side not specified), symptomatic intracranial hemorrhage, Follow-up >1 year.	USA	High risk	Serious risk of bias (Confound ing, Selection, Informatio n, Reporting)
Markus 2019	MIX	Randomiz ed, prospectiv e, open label, internation al, multicenter Study	197 Patients with Cervical Artery Dissection, 98 Patients with eICAD, 151 with Stroke, 35 with TIA, 136 with Headache, 92 with Neck Pain, 53 with Horner's, 1 with Retinal Infarction. Mixed etiology.	47 anticoagulants, 51 antiplatelets	Death, Stroke (ipsilateral), TIA, Major Bleeding. Outcomes were assessed at 12 month.	UK, Australia Additional information obtained through personal communication	Table S6	
Marx 1987	PUB	Observatio nal study on	8 patients with eICAD, 4 with stroke, 2 with	6 anticoagulants, 1 antiplatelet	Death, disability, stroke (ischemic, hemorrhagic, side not specified)	Germany	High risk	Serious risk of bias (Confound

For Peer Review

		spontaneous dissections of cervical arteries	Horner's syndrome, 2 TIA	drugs, 1 no treatment				ing, Selection, Information, Reporting)
Metso 2009	MIX	Observational study, single center, consecutive patients with cervico-cerebral artery dissection	301 patients with either extra or intracranial internal carotid or vertebral artery dissection. Spontaneous. Data about outcome stratified to the type of antithrombotic treatment were obtained by personal communication	For the 144 eICAD patients: 140 anticoagulants, 4 antiplatelets	Death, dependency, stroke (side not specified), TIA, symptomatic intracranial hemorrhage. Follow-up at 6 months.	Finland, Additional information obtained by personal communication, in 1 of the 140 patients treated with anticoagulation there was no information on disability available, Data overlap with Traenka 2020 for the outcomes of death, death or disability and ischemic stroke	High risk	Serious risk of bias (Confounding, Selection, Information, Reporting)
Miller-Fisher 1978	PUB	Observational study on spontaneous dissections of cervical arteries	16 patients with eICAD, 10 with TIA, 0 with stroke, 10 with pain. Spontaneous.	2 anticoagulants, 1 antiplatelet drugs, 5 surgery (3 explorations, 2 embolectomies) 8 not	Death, disability, stroke (side not specified), symptomatic intracranial hemorrhage. Follow-up >1 year.	USA, Information on treatment for 8 patients not given	High risk	Serious risk of bias (Confounding, Selection, Information, Reporting)

For Peer Review

				mentioned				
Mokri 1986	PUB	Observational study, retrospective assessment	36 patients with eICAD, 30 with headache, 2 with stroke, 2 with TIA or AF, 1 syncope, 1 neck pain. Spontaneous.	10 anticoagulants, 9 antiplatelet drugs, 14 no treatment, 1 surgery (EC/IC bypass), 3 not mentioned	Death, disability, stroke (ischemic, hemorrhagic, side not specified), TIA, Follow-up >1 year.	USA, Information on initial therapy not reported in 2; 6 patients had EC/IC bypass surgery later	High risk	Serious risk of bias (Confounding, Selection, Information, Reporting)
Müller-Forell 1989	PUB	Observational study, angiographical and ultrasound diagnosis	4 patients with eICAD, 2 with stroke, 2 with pain. Spontaneous.	1 anticoagulants, 1 antiplatelet drugs, 1 no treatment, 1 not mentioned	Death, stroke (side not specified).	Germany, Initial therapy not reported in 1 patient	High risk	Serious risk of bias (Confounding, Selection, Information, Reporting)
Pieri 2007	MIX	Observational study, case series	66 patients with cervical artery dissection, 24/66 had eICAD. Spontaneous.	10/24 had antiplatelets, 14/24 had anticoagulation	Death, disability, stroke (ischemic, hemorrhagic, side not specified), symptomatic intracranial bleedings, TIA. Follow-up at 6 months.	Sao Paulo, Brazil, Additional information obtained by personal communication	High risk	Serious risk of bias (Confounding, Selection, Information, Reporting)
Reges 2019	SOUGHT	Observational, retrospective, monocentric	41 patients with cervical artery dissections, 18/41 had eICAD. Spontaneous.	13/18 had antiplatelets, 5/18 had anticoagulation	Death, stroke (side not specified), intracranial heamorrage, modified ranking scale and recanalization. Follow-up at 6 months.h	Brasil, Unpublished data on stroke and symptomatic intracranial bleeding sought, but not provided	High risk	Serious risk of bias (Confounding, Selection, Information, Reporting)

For Peer Review

Richaud 1980	PUB	Observational study, case series from trauma patients	17 patients with eICAD, 9 with stroke, 2 with TIA (information or other patients not conclusive). Traumatic.	5 anticoagulants, 1 antiplatelet drugs, 4 no treatment, 3 surgery (non-arterial operations), 4 not mentioned	Death, disability, stroke (side not specified).	France, Confounding from trauma possible, No report on initial therapy in 4 patients	High risk	Serious risk of bias (Confounding, Selection, Information, Reporting)
Schievink 1990	PUB	Observational study, consecutive case series	7 patients with eICAD, 3 with stroke, 4 with local symptoms. Mixed etiology.	2 anticoagulants, 5 antiplatelet drugs	Death, disability, stroke (side not specified)	The Netherlands	High risk	Serious risk of bias (Confounding, Selection, Information, Reporting)
Sellier 1983	PUB	Observational study, case series on spontaneous eICAD, traumatic cases excluded	46 patients with eICAD, 18 with stroke, 24 with TIA. Spontaneous.	16 anticoagulants, 13 antiplatelet drugs, 10 no treatment, 7 surgery (intervention not specified)	Death, stroke (ischemic, hemorrhagic, side not specified)	France	High risk	Serious risk of bias (Confounding, Selection, Information, Reporting)
Sercl 2020	MIX	Retrospective, momocentric observational study	30 eICAD patients, 13 traumatic. Mixed etiology.	7 patients anticoagulants, 13 antiplatelets, 8 stenting after tPA, 2 no further treatment after tPA	Death, disability, stroke (side not specified), TIA, symptomatic intracerebral heamorrhage and major extracranial haemmorhage at 3 month, Follow-up >1 year.	Poland, Delayed stenting in 2 patients under antiplatelet therapy (6 & 8 month respectively), thus not	High risk	Serious risk of bias (Confounding, Selection, Information, Reporting)

For Peer Review

						excluded fron data analysis, Additional information obtained through personal communication		
Touze 2003	PUB	Multicenter observational study, databank-based, 24 neurology departments	Consecutive patients with cervical artery dissection (eICAD or eVAD or both), The data for the subgroup of patients with eICAD alone were obtained by personal communication. Mixed etiology.	For the 311 'eICAD alone' patients: 279 anticoagulants; 18 antiplatelets, 12 no antithrombotics, 2 tPA	Stroke (ipsilateral), TIA, death, recurrent dissection. Follow-up >1 year.	France, Additional information obtained by personal communication	High risk	Serious risk of bias (Confounding, Selection, Information, Reporting)
Traenka 2020	MIX	Multicentric, Observational study, Dissection registry based	1382 Patients with eICAD. Mixed etiology.	855 received Anticoagulants, 452 received Antiplatelets, 5 had no treatment, or not defined or missing	Functional outcome at 3 and/or 6 months, Stroke (side not specified), recurrent dissection, major hemorrhage, death	Europe, USA, Additional information obtained by personal communication Data on Ischemic Stroke missing from 7 Anticoagulant	High risk	Serious risk of bias (Confounding, Selection, Information, Reporting)

For Peer Review

						Patients and 16 Antiplatelet Patients. Data on Intracranial and Major Extracranial Hemorrhage pooled.		
Treiman 1996	PUB	Observational case series	24 patients with eICAD, diagnosis made by angiography. Spontaneous.	3 no antithrombotic treatment (died), 21 patients who survived the initial hospitalisation, 12 anticoagulation, 6 antiplatelets, 3 with combined antiplatelets plus anticoagulation	Vascular death, stroke (side not specified), recurrent dissection, bleeding complication. Follow-up >1 year.	USA, No information about type of initial antithrombotic treatment available in 2 patients with non-vascular death years after the eICAD	High risk	Serious risk of bias (Confounding, Selection, Information, Reporting)
Vanneste 1984	PUB	Observational study, selection not reported	5 patients with eICAD, 2 with stroke, 3 with pain. Spontaneous.	4 anticoagulants, 1 antiplatelet drugs	Death, disability, stroke (side not specified), symptomatic intracranial bleeds. Follow-up >1 year.	The Netherlands	High risk	Serious risk of bias (Confounding, Selection, Information, Reporting)
Vineetha 2019	MIX	Observational, prospectiv	132 Patients with eICAD. Spontaneous.	39 patients receiving anticoagulation	Stroke (ipsilateral), TIA, symptomatic intracranial hemorrhage, disablity, major	IndiaAdditional information obtained	High risk	Serious risk of bias (Confound

For Peer Review

		e, single center		and 93 receiving antiplatelts	extracranial hemorrhage, death. Follow-up at 3 and/or 6 months.	through personal communication		ing, Selection, Informatio n, Reporting)
Wahl 2002	PUB	Observatio nal study, traumatic carotid dissections (i.e. dissection of common carotid artery, eICAD, and intracranial dissections , trauma center registry)	11 patients with traumatic eICAD (among 22 patients with carotid dissections of any kind). Traumatic.	In subgroup of 11 patients with eICAD: 7 anticoagulants, 1 antiplatelets only, 1 no antithrombotic agents, 2 stents	Death, disability, stroke (side not specified), major extracranial hemorrhage	USA, Confounding of outcome assessment with trauma possible	High risk	Serious risk of bias (Confound ing, Selection, Informatio n, Reporting)
Zelenock 1982	PUB	Observatio nal study, case series of trauma patients	6 patients with eICAD. Traumatic.	1 anticoagulants, 1 antiplatelet drugs, 3 no treatment, 1 surgery (ligation)	Death, disability, stroke (ischemic, hemorrhagic, side not specified). Follow-up >1 year.	USA, Confounding with trauma possible	High risk	Serious risk of bias (Confound ing, Selection, Informatio n, Reporting)

Table S6: Risk of Bias of randomized studies.

Engelter 2021		
**Risk of Bias Domains**	**Judgement**	**Reason**
Random sequence generation (selection bias)	Low risk	"Randomisation was computer-generated, using an interactive web response system, with stratification according to the participating sites. The randomisation sequence was generated by members of the Clinical Trial Unit of the University Hospital Basel (Basel, Switzerland)."
Allocation concealment (selection bias)	Low risk	There was no selection bias due to the concealment of treatment allocation.
Blinding of participants and personnel (performance bias): All outcomes	High risk	"investigators, patients, and the independent clinical event adjudication committee members (appendix 1 p 2) were aware of treatment allocation"
Blinding of outcome assessment (detection bias): All Outcomes	Low risk	"Independent imaging core laboratory adjudicators (Clinical Stroke & Imaging Analysis Lab Basel, University of Basel, Basel, Switzerland; appendix 1 p 2) were masked to the allocated treatment"
Incomplete outcome data (attrition bias): All Outcomes	Low risk	There was only a small number of missing follow up data in the per protocol population
Selective reporting (reporting bias)	Low risk	All prespecified primary and secondary outcomes were reported.
Other bias	Low risk	No other bias identified.

For Peer Review

Markus 2019		
**Risk of Bias Domains**	**Judgement**	**Reason**
Random sequence generation (selection bias)	Low risk	"Randomization was provided via an automated 24-hour telephone randomization service provided by the University of Aberdeen, Scotland."
Allocation concealment (selection bias)	Low risk	Thera was no selection bias due to the process fo treatment allocation
Blinding of participants and personnel (performance bias): All outcomes	High risk	"both patients and clinicians were aware of treatment allocation"
Blinding of outcome assessment (detection bias): All Outcomes	Low risk	"However, an adjudication committee assessed all primary end points (stroke) and secondary end points blinded to treatment arm."
Incomplete outcome data (attrition bias): All Outcomes	Low risk	Follow-up to 12 months was obtained in all patients.
Selective reporting (reporting bias)	Low risk	All prespecified primary and secondary outcomes were reported
Other bias	Low risk	No other bias identified

For Peer Review

Table S7: Excluded studies with reasons for exclusion.

Study	Reason for exclusion
Ahmad 1999	No treatment with antiplatelet drugs
Alimi 1996	No treatment with antithrombotic drugs<br>Observational study
Almendrote 2015	No outcome separation between eICAD and VA Dissection No information for antiplatelet drugs<br>Retrospective observational study
Andre-Sereys 1996	No treatment with antiplatelet drugs<br>Observational study
Arnold 2006	No information on outcome stratified by type of antithrombotic treatment<br>Outcome not stratified by type of affected cervical artery dissection (i.e. eICAD and vertebral artery dissection reported together)<br>Gender differences was primary objective<br>Overlap with included study Georgiadis 2009; the latter focused on treatment and outcome
Bakke 1996	No treatment with anticoagulants<br>Observational study
Barbour 1994	No information on management and outcome
Barrachini 2010	No patients treated with antiplatelets<br>All patients received Heparin<br>Observational Study
Bassetti 1996	No information on treatment<br>No separation between dissection of ICAD and VA
Bassi 2003	Outcome information not stratified to treatment<br>Observational study
Baumgartner 2001	No information on treatment
Berne 2004	None of the eICAD patients was treated with antiplatelet agents alone
Biffl 2002	No separation between dissection of ICAD and VA<br>No separation of traumatic eICAD from patients with carotid injury other than dissection<br>All trauma patients<br>Observational study
Biousse 1995	Information on outcomes not available in respect to treatment
Blitzer 2020	No information on treatment with antiplatelet or anticoagulant drugs
Bradac 1981	No treatment with antithrombotic drugs<br>Observational study
Bui 1993	Diagnostic study, no outcome information

Burlew 2018	No outcome and treatment related stratification between eICAD and VA<br>Dissections Only patients with stroke<br>Multicenter retrospective study
Caprio 2014	No infomration regarding primary outcome<br>No outcome-related separation between ICAD and VA<br>Observational Study
Carrillo 1999	No separation between dissections of ICA and those of the CCA
Catapano 2020	No outcome separation between eICAD and VA<br>Dissections Mixed Patients with antiplatelet and anticoagulation treatment<br>No outcomes on patients with anticoagulation<br>Retrospective observational study
Chabrier 2003	Outcome not stratified by type of involved artery
Chan 2001	No treatment with antiplatelets drugs<br>All patients treated with anticoagulants
Chandra 2007	No patients treated with antiplatelet drugs<br>Retrospective observational study
Cimini 2004	Outcome not stratified to type of treatment
Cogbill 1994	No patients treated with antiplatelet drugs
Crawford 2015	No outcome and treatment type related stratification between eICAD and VA dissections<br>Retrospective observational study
Crespo Araico 2019	No information about antiplatelet or anticoagulant drugs<br>Only patients with intravenous thrombolysis and intervention<br>Retrospective observational study
Cusmano 1988	Treatment only with antiplatelet drugs<br>Observational study
Daou 2017	No outcome related separation between dissection of ICA and VA<br>Observational study
Davis 1990	Treatment only with anticoagulants<br>Observational study
Desfontaines 1995	Study Reason for exclusion<br>Outcome information not stratified to treatment<br>Observational study
DiCocco 2011	No outcome separation between medical and interventional treatment<br>Outcome information not stratified to treatment type<br>Observational study
Djouhri 2000	No information on treatment<br>Diagnostic study (MRA)<br>Overlapping with included study Biousse 1998 and excluded studies Biousse 1995 and Guillon 1999
Dreier 2004	Treatment only with anticoagulants
Droste 2001	Diagnostic study (transcranial doppler sonography)<br>'Recurrent ischemic events' not specified

For Peer Review

Early 1991	Treatment only with anticoagulants<br>Observational study
Ehrenfeld 1976	No treatment with antithrombotic drugs<br>Observational study
Engelter 2012	Overlapping with included study Traenka 2020
Fabian 1990	No treatment with antiplatelet drugs<br>Observational study
Fabian 1996	No separation of ICA dissection from ICA thrombosis or carotid cavernous fistulas
Figueroa 2021	No differentiation between extracranial and intracranial ICA injury<br>No clear differentiation between ICA dissections and other injury types<br>Observational Study
Franz 2010	No differentiation between CCA & ICA dissection<br>Incomplete follow-up
Fuentes 2011	No information on therapy with Antiplatelets or Anticoagulants<br>Multicenter Observational Study
Gelbert 1991	No information on therapy
Guillon 1999	No antiplatelet therapy<br>Overlapping with included study Biousse 1998and excluded studies Biousse 1995 and Djouhri 2000<br>All patients included had aneurysms (observational study)
Harrigan 2020	No patients treated with anticoagulant drugs<br>Prospective observational study
Haussen 2015	No information on treatment with antiplatelet or anticoagulant drugs<br>Patients treated only with intervention<br>Retrospective observational study
Hughes 2000	No separation between extra and intracranial ICA dissection<br>Observational study<br>Only trauma patients
Jensen 2017	No information on patients treated with antiplatet or anticoagulant drugs<br>Only patients with stroke included<br>Retrospective observational study
Jones 2012	No information of patients treated with antiplatelet or anticoagulant drugs<br>No outcome and treatment related separation between eICAD and VA dissections<br>Pediatric population<br>Observational Study
Kelly 2014	Case series with less than 3 Patients with eICAD<br>Observational Study
Kerwin 2001	No outcome and therapy-related separation between A: eICAD and vertebral artery dissection B: eICAD and other non-dissection ICA injuries (e.g. carotid-cavernous sinus

For Peer Review

	fistula)<br>All patients had trauma
Kirsch 1998	No information on treatment<br>Diagnostic study (MRA)
Koennecke 1997	No treatment with antiplatelet drugs<br>No follow-up information
Landini 1996	No treatment with anticoagulants<br>Observational study
Larsson 2017	Data included in Kennedy 2012 and Markus 2019
Laser 2017	Only CCA dissections<br>Observational study
Le 2020	No outcome and treatment-type related differentiation between eICAD and VA Dissection
Leclerc 1998	Diagnostic study (CT follow up)<br>No treatment with antiplatelet drugs<br>Overlapping with excluded study Leys 1995
Lee 2006	Information on outcomes not available in respect to treatment and affected artery<br>Epidemiological, population-based study
Leichtle 2020	No outcome and treatment related stratification between eICAD and VA dissections<br>Observational Study
Leys 1995	No separation between ICAD and VA dissection
Leys 1997	Review Article
Lisovoski 1991	No information on management
Lucas 1998	No information on therapy
Machet 2013	No outcome and treatment related stratification between eICAD and VA dissection<br>Only radiographic outcomes<br>Observational study
Markus 2015	Data included in Markus 2019
Miller 2001	No outcome and therapy-related separation between A: eICAD, CCA and VA dissection B: eICAD and other non-dissection ICA injuries (e.g. carotid-cavernous sinus fistula)<br>All patients had trauma
Mokri 1990	Information on outcomes not available in respect to treatment<br>Confounding with included study Mokri 1986 possible
Molina 2000	No separation between extra and intracranial ICA dissection<br>All patients had anticoagulation<br>Diagnostic study (emboli detection by transcranial doppler sonography)
Morgan 1994	No treatment with antiplatelets<br>Observational study
Mueller 2000	Study on surgery for eICAD (all patients had surgery)
Mustanoja 2015	No patients treated with antiplatelet drugs<br>Comparison between vitamin K antagonists and direct oral

For peer review only

	anticoagulants<br>Observational Study
Müllges 1992	No treatment with anticoagulants<br>Observational study
Nishino 2008	Outcome not stratified to involved artery and treatment<br>Observational study
O'Dwyer 1980	No separation between ICAD and CCA dissection<br>Observational study
Oliveira 2001	Outcome not stratified to treatment<br>Diagnostic study (transcranial doppler emboli monitoring)<br>Observational study
Ota 2019	Only 3 Patients with ICAD Patients with T and non-T ICA occlusions<br>No outcome separation between treatment with antiplatelet or anticoagulant drugs<br>Observational study
Parenti 1989	No treatment with anticoagulants<br>Observational study
Pelkonen 2003	Outcome information not stratified to treatment<br>Observational study
Perry 1980	No treatment with antithrombotic drugs<br>Observational study
Petro 1987	No treatment with antiplatelet drugs<br>Observational study
Pezzini 2022	No outcome and treatment related stratification between eICAD and VA dissection<br>Observational Study
Power 1991	No treatment with antiplatelet drugs<br>Observational study
Pozzati 1990	No treatment with antithrombotics<br>Observational study
Pretre 1994	No treatment with antiplatelet drugs<br>Observational study
Provenzale 1995	No information on management<br>Observational study
Qureshi 2011	No outcome and treatment related stratification between eICAD and VA dissection<br>Observational Study
Ramchand 2018	No outcome and therapy-related separation between dissection of ICA and VA<br>Single-center retrospective cohort study
Rosati 2020	No outcome and therapy-related separation between extracranial and intracranial ICAD, VA and basilar artery<br>Observational Study
Schievink 1994	No information on management<br>Observational study

For Peer Review

Schwartz 2009	No outcome and therapy-related separation between ICAD and VA<br>Observational study, database
Scott 2015	No patients with only eICAD treated with anticoagulant drugs<br>Observational Study
Simonetti 2019	Only 3 Patients with eICAD treated with antiplatelet or anticoagulation therapy without intervention<br>Iatrogenic Dissection<br>Only oral anticoagulants in the anticoagulant group<br>Prospective database
Sperling 1996	No treatment with antiplatelet drugs<br>Observational study
Steiger 1988	No treatment with antiplatelet drugs<br>Observational study
Steinke 1994	No treatment with antiplatelet drugs<br>Observational study
Stringer 1980	No treatment with antiplatelet drugs<br>Observational study
Sturzenegger 1995	Data included in Georgiadis 2009
Sue 1992	No treatment with antiplatelet drugs<br>Observational study
Tansirisithikul 2013	No outcome separation between treatment types<br>Observational study, registry-based
Thie 1993	Missing information on treatment and outcomes
Touze 2001	No information on treatment<br>No separation between dissections of ICA and those of VA
Van Damme 1990	No treatment with anticoagulants
Verdalle 2001	No treatment with antiplatelets
Vishteh 1998	No separation between extracranial ICAD and intracranial ICA pathologies (e.g. cavernous ICAD, carotid cavernous fistula)
Watridge 1989	Confounding with data from excluded study Fabian 1990 possible
Welling 1987	Patients included in the excluded Study Welling 1989<br>No patients treated with antiplatelet drugs<br>Observational study
Welling 1989	No treatment with antiplatelet drugs<br>Observational study
Yaghi 2012	No outcome and therapy-related separation between ICAD and VA Observational Study

For Peer Review
